# Repression/Depression of Conjugative Plasmids and Their Influence on the Mutation-Selection Balance in Static Environments

**DOI:** 10.1371/journal.pone.0096839

**Published:** 2014-05-08

**Authors:** Yoav Raz, Emmanuel David Tannenbaum

**Affiliations:** Department of Chemistry, Ben-Gurion University of the Negev, Beér-Sheva, Israel; Centre National de la Recherche Scientifique, Aix-Marseille Université, France

## Abstract

We study the effect that conjugation-mediated Horizontal Gene Transfer (HGT) has on the mutation-selection balance of a population in a static environment. We consider a model whereby a population of unicellular organisms, capable of conjugation, comes to mutation-selection balance in the presence of an antibiotic, which induces a first-order death rate constant 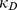 for genomes that are not resistant. We explicitly take into consideration the repression/de-repression dynamics of the conjugative plasmid, and assume that a de-repressed plasmid remains temporarily de-repressed after copying itself into another cell. We assume that both repression and de-repression are characterized by first-order rate constants 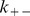
and 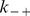, respectively. We find that conjugation has a deleterious effect on the mean fitness of the population, suggesting that HGT does not provide a selective advantage in a static environment, but is rather only useful for adapting to new environments. This effect can be ameliorated by repression, suggesting that while HGT is not necessarily advantageous for a population in a static environment, its deleterious effect on the mean fitness can be negated via repression. Therefore, it is likely that HGT is much more advantageous in a dynamic landscape. Furthermore, in the limiting case of a vanishing spontaneous de-repression rate constant, we find that the fraction of conjugators in the population undergoes a phase transition as a function of population density. Below a critical population density, the fraction of conjugators is zero, while above this critical population density the fraction of conjugators rises continuously to one. Our model for conjugation-mediated HGT is related to models of infectious disease dynamics, where the conjugators play the role of the infected (I) class, and the non-conjugators play the role of the susceptible (S) class.

## Introduction

Horizontal Gene Transfer (HGT) is defined as the transfer or exchange of genetic information between two organisms, that does not involve the transmission of genetic information from parent to daughter as a result of replication, a process known as *vertical transmission of genetic information* [1], [2]. Since it was initially characterized in the 1940s [3], it has gradually become evident that HGT is one of the major sources of genomic change in prokaryotes [2]. As a result of its recognized importance for shaping the genomes of prokaryotes, a complete theory describing the evolutionary dynamics of prokaryotes must take HGT into account [2]. Further, the fact that HGT plays a major role in shaping prokaryotic genomes means that HGT must be considered when reconstructing phylogenetic trees [1], which of course complicates any effort to understand the evolutionary relationships amongst bacterial strains, and to trace the evolutionary histories of various genes. The existence of HGT also has consequences for public health, since HGT is believed to be primarily responsible for the rapid spread of antibiotic drug resistance in bacterial populations [2], [4]–[8].

Despite the large influence that HGT has on the evolutionary dynamics of prokaryotic populations [2], with a few notable exceptions [9]–[12], there has been relatively little theoretical work attempting to incorporate HGT into evolutionary models. Indeed, it is safe to say that the bulk of the work in evolutionary biology has focused on point-mutations as the primary source of genetic variation [13]. There are two main reasons for this: First of all, the simplest evolutionary models are the ones that consider point-mutations as the only source of genetic variation. Yet even these models can exhibit fairly non-trivial behavior, and there is a wide range of additional effects that may be considered within the point-mutation framework (e.g., conservative versus semiconservative replication, fitness landscapes, polysomic genomes, genetic instability, genetic repair and mutators, finite-size effects [9], [13]–[21]). Thus, already at the simplest level of point-mutations, there is a fairly large class of evolutionary models that may be analyzed. Second, for quite some time in biology it was believed that point-mutations are indeed the primary source of genetic change in organisms. This assumption underlies the concept of the so-called “tree of life” that was used to organize phylogenetic data. With the realization of the importance of HGT, there have been suggestions to replace the tree analogy with that of a web or a ring [1], [22]–[25].

Bacterial genomes typically consist of a single, large, circular chromosome, in addition to several smaller, circular chromosomes known as plasmids. Bacterial plasmids can move between bacteria, often between bacteria of different strains, via a process known as *conjugation*.[26]–[29]. If these plasmids contain genes for an adaptive trait such as antibiotic drug resistance, then conjugation can significantly speed up the rate at which such an adaptive trait spreads throughout a population.[30]–[33]. Conjugation is believed to be one of the most important forms of HGT, so that both phenomenological and quantitative models of processes such as the emergence of antibiotic drug resistance will need to properly account for this behavior.

**Figure 1:**
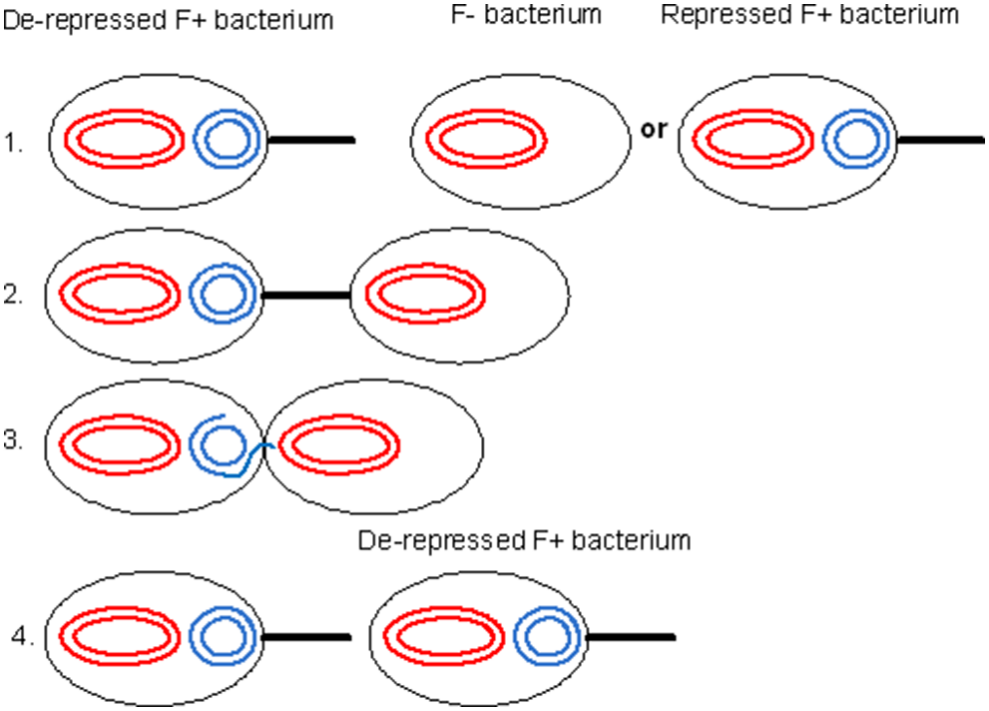
The conjugation process for F+ bacterium with F- bacterium as detailed in our model: 1. A de-repressed bacterium comes within proximity of an F- bacterium/repressed F+ bacterium. 2. The F+ bacterium’s pili interacts with the F- bacterium’s membrane. 3. The F+ plasmid releases a strand of its DNA to the F- bacterium via conjugation machinery. 4. A new de-repressed F+ bacterium is formed.

The best characterized bacterial conjugation system is the F^+^/F^−^ system [28]. Here, a bacterium containing what is termed an F-plasmid fuses with a bacterium lacking the F-plasmid. The bacterium containing the F-plasmid is termed an F^+^ bacterium, while the bacterium that does not contain this plasmid is termed an F^−^ bacterium. When an F^+^ bacterium meets an F^−^ bacterium, it transfers one of the strands of the F-plasmid to the F^−^ bacterium via a pilus. Once a strand of the s F-plasmid has been transferred from the F^+^ bacterium to the F^−^ bacterium, a copy of the plasmid in both cells is produced by daughter strand synthesis using the DNA template strands. The F^−^ bacterium then becomes an F^+^ bacterium that transcribes its own pilus, and is able to transfer the F^−^plasmid to other bacteria in the population (Figure 1).

The F^+^/F^−^ system is in some ways atypical for bacterial conjugation systems, due to its conjugative machinery being chronically in a de-repressed state, while in nature most conjugative plasmid systems are repressed via the expression of T4SS modules [34], [35]. Nevertheless, the F-plasmid system is still one of the best characterized bacterial conjugation systems, and it can be considered as broadly representative of all known bacterial conjugation systems. Therefore, it makes sense to base mathematical models of conjugation-mediated HGT on the F-plasmid system.

**Figure 2:**
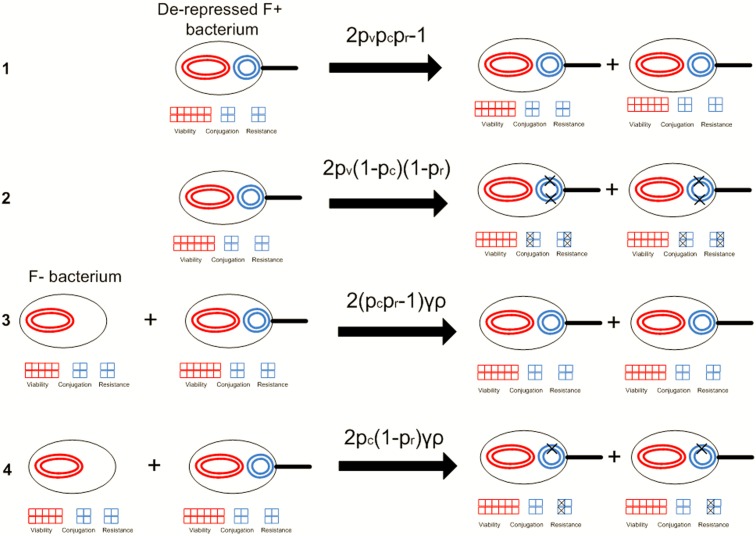
Examples for vertical replication and horizontal gene transfer within the framework of our model and the consequential propogation of mutations throughout the population: 1. Mutation-free vertical replication of the de-repressed, fully viable subpopulation that is notated as 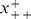
_._ 2. Vertical replication followed by a mutation to the conjugation and antibiotic resistance genes in the plasmid. 3. Mutation-free bacterial conjugation between an F+ and an F- bacterium that leads to the formation of two antibiotic resistant, conjugation capable, derepressed bacteria. 4. Horizontal transfer between an F+ and F- bacterium followed by a mutation to the antibiotic resistance gene of both conjugants, leading to no drug resistance for these bacteria. 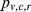 respectively notate the probabilities for the bacterium replicating its viability, conjugation, and resistance portions of the genome in an error-free manner while 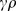 is the second order reaction parameter of the conjugation rate.

In recent work [36], the authors developed mathematical models describing the role that conjugation has on the mean fitness of unicellular populations in static environments, and found that conjugation-mediated HGT does not confer a selective advantage to the bacterial population (indeed conjugation-mediated HGT was found to have a slightly deleterious effect on the mean fitness of a population). However, in this work, it was assumed that the conjugation machinery is permanently de-repressed. Here, we extend our previous work by introducing the repression/derepression mechanism into our original model [13] of a bacterial population that transfers genetic information among its members both vertically and horizontally (Figure 2).

## Results

### 1. Definition of the Model

Our model assumes a unicellular population of asexually reproducing bacteria, where the bacterial genome consists of two chromosomes. The first chromosome is the main circular bacterial chromosome that transfers its genetic information via vertical transmission (i.e. replication from one generation to the next). The second chromosome is taken to be the much smaller conjugative plasmid, which is assumed to consist of two regions: (1) A “conjugation” region, comprising the various genes necessary for bacterial conjugation. (2) An “antibiotic resistance” region, comprising the genes necessary for conferring resistance to a given antibiotic. Since each chromosome is assumed to be a double-stranded, semiconservatively replicating DNA molecule, we define probabilities for the error-free replication of a template strand from each of the various regions of the bacterial genome. To this end, we let 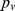 denote the probability that a template strand from the main bacterial chromosome produces a daughter chromosome without introducing any new point-mutations. We define 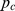 and 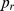 analogously for the portions of the bacterial plasmid coding for conjugation and antibiotic drug resistance. We assume that there are master sequences for the main bacterial genome, the conjugation and antibiotic drug resistance regions of the plasmid. We assume that any mutation to these master sequences renders the sequences non-functional. We further assume that the bacterial genome controls viability, and so a bacterium with a master, or wild-type, copy of its main chromosome, has a normalized first-order growth rate constant of 1, while a bacterium with a mutated main chromosome has a first-order growth rate constant of 0. The assumption that a single mutation renders the master sequence non-functional is known as the single-fitness-peak approximation [13]. Although it is an oversimplification of the true dependence of functionality on the gene sequence, it nevertheless captures the fact that only a small fraction of sequences are likely to yield a gene that encodes the desired function. As a result, this approximation has been known to give quantitative results in certain cases [37].

Given its simplicity, it therefore makes sense to start with this assumption before moving on to more complicated fitness models. We assume that a viable bacterium whose resistance region of the plasmid corresponds to the wild-type is unaffected by the presence of an antibiotic, while a viable bacterium with a mutated portion of this resistance region is killed by the antibiotic at a rate characterized by a first-order rate constant 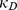.

Bacterial conjugation is modeled as a second-order rate process characterized by a rate constant γ. We also assume that, as the size of the bacterial population changes, the system volume changes so as to maintain a constant population density ρ while assuming that ρ remains constant. It should be noted that in this context, the only bacteria whose population we are tracking are the viable ones. The idea behind this assumption is that the bacteria each take up a certain amount of space, and so, as the bacterial population grows, the total system volume must increase as well. Bacterial conjugation can only occur between a viable bacterium whose conjugation genes are de-repressed, and a viable bacterium whose conjugation genes are repressed. The repression/de-repression dynamics is assumed to be characterized by first-order rate constants 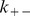
and 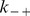, respectively, corresponding to transitions from the de-repressed to the repressed state, and from the repressed state to the de-repressed state, respectively. However, for the purposes of this paper, we will assume that 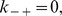 corresponding to the limit of weak de-repression, which is believed to characterize the repression/de-repression dynamics of actual systems [34], [38].

During bacterial conjugation, we assume that a template strand of the plasmid from the de-repressed bacterium, which is labelled the “+” bacterium, is transferred to the repressed, or “−”, bacterium, and that daughter strand synthesis occurs in both bacteria to reconstitute the plasmids. We assume that the plasmid transferred from the “+” bacterium replaces the plasmid in the “−” bacterium, and inherits the de-repressed state of the parent plasmid. The assumption of plasmid replacement is a simplification that will need to be re-examined in future research, where we anticipate developing more accurate models that allow for variable plasmid numbers in the bacterial cell. The basis for this assumption derives from the observation that plasmids of similar compatibility classes cannot co-exist in the same cell [39], [40], and that bacteria can control the number of plasmids in the cell [40]–[42]. While these observations do not directly translate into a plasmid replacement model, we believe that they do provide some justification for this assumption.

Finally, we assume that replication errors are due to mismatches during daughter strand synthesis that are sub-sequentially fixed in the genome. We let ε denote the mismatch probability per base-pair, so that ε/2 is the probability of making a mismatch, which is fixed as a mutation in the genome. If 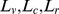 denote the lengths of the viability, conjugation, and resistance portions of the genome, respectively, then we have that 
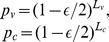
 and 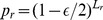.

Now, following the standard procedure in population genetics and quasispecies models [13], [19], we may allow the genome length 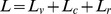 to become very large while holding 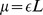 constant. Defining 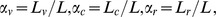 we then obtain,
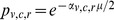
(1)


### 2. Evolutionary Dynamics Equations

Here, we define 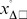 to be the fraction of the population consisting of viable bacteria such that the conjugator region is of type △, and the resistance region is of type □, where a type of “+” means that the given region is of the wild-type, while a type of “−” means that the given region differs from the wild-type. When △ = +, corresponding to a conjugator, then ° = ±, corresponding to the genes for conjugation being de-repressed (° = +) or repressed (° = −). Since we are only tracking viable bacteria, we should note that 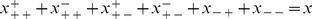
_._

We define 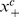 to be the total fraction of viable bacteria that are conjugators, so that 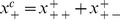, and we define 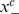 to be the total fraction of viable bacteria that are non-conjugators, so that 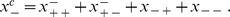 Therefore, allowing us to summarize all of the processes that are relevant for each subpopulation in our model into a generalized list of balance equations which are described by Eq. (2):
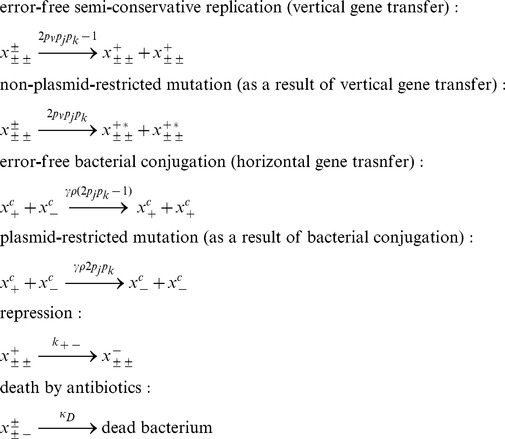
(2)


We note that 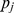 denotes two possible values for the conjugation gene - 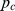 when the gene is copied or conjugated in an error-free manner, or 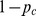 when the gene is copied incorrectly. In a similar manner, 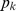 denotes two possible values for the antibiotics resistance gene - 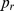 or 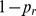.

Putting everything together, we obtain that the evolutionary dynamics of the bacterial population is governed by the following system of equations:
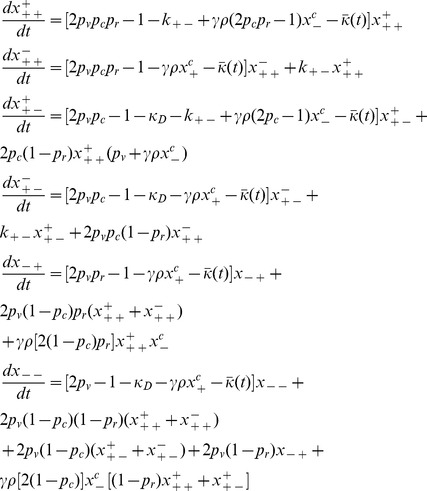
(3)


By summarizing all of these equations we get that 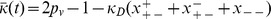 is the mean fitness of the population, or the first-order growth rate constant of the population as a whole. Finally, we’d also like to note that the form of the equations detailed by Eq. (3) is identical to what would be obtained if we made a more realistic assumption that the population was growing in a chemostat. [13].

### 3. Mutation-selection Balance

We solve our system of equations for the mutation-selection balance for the case when µ is small enough so to allow us to avoid the error-catastrophe region. This allows us to apply a first order taylor expansion to Eq. (1), which gives us simpler terms for 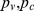 and 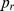 which are 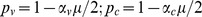 and 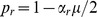 (the probabilities for an error-free replication of bacterium’s chromosome, conjugation gene and resistance gene respectively). Our first goal is to determine the initial conditions which will allow our system to converge while maintaining physical context. We proceed by considering a preliminary condition for the antibiotics-related death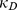 which must be greater then the value of zero while denoting 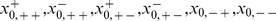 as the 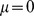 values of 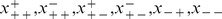, respectively. We show in sub-section A from the Materials and Methods that this condition brings forth that there are no non-resistant organisms in the system when µ = 0 so that 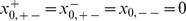. From this point, we separate our analysis into two regions based on the differences between the values of
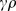
and 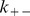. The first region obeys the condition of 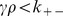 and we have proved that for this region 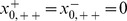
and 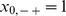
(sub-section B from the Materials and methods). The second region obeys the condition of 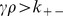 and we proved that for this region 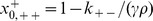,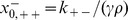,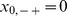 (sub-section C of the Materials and methods). We applied these to Eq. (3) and found two separate solutions for 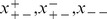 and consequently were able to compute the terms for the population mean fitness for each of these regions (sub-section D in Materials and methods). For 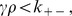 we have 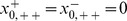,and 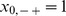 while 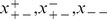 have the following values:
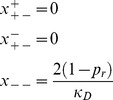
(4)


Therefore, in selection-mutation balance their population mean fitness is:
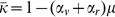
(5)


The second solution is for 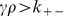 when 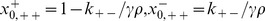 and 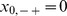 when we obtain,
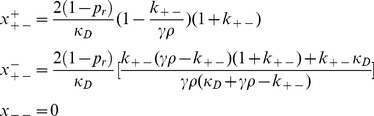
(6)


from which we derive the selection-mutation balance mean fitness of:
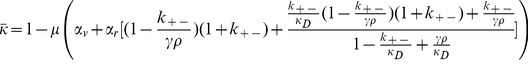
(7)


**Figure 3:**
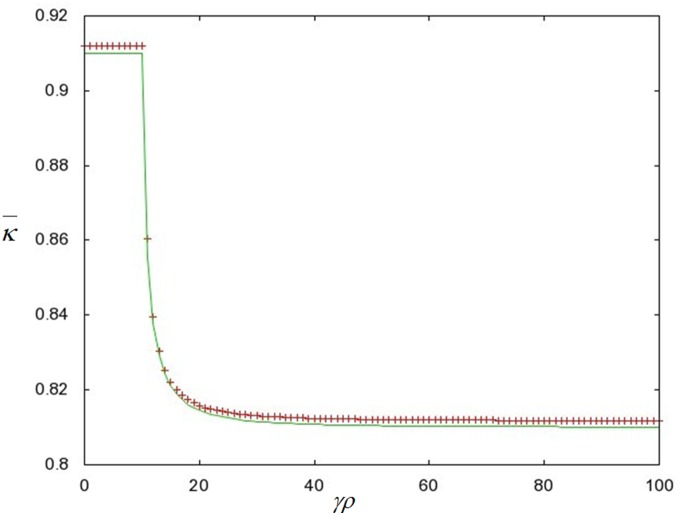
A plot of 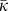 (the population mean fitness) versus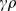 (the second order reaction parameter of the conjugation rate). Parameter values are
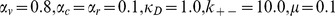
(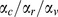 are the ratio of the genome which is dedicated to conjugation/resistance/organism viability respectively, 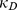 is the first-order death rate constant of the population that is caused by antibiotics, 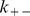 is the first order rate constant which describes the conjugators transition from the de-repressed to the repressed state and µ describes the probability of mutation for every replication of the entire genome). The solid line represents the analytical expression for 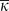 obtained in the small-µ limit, while the pluses represent the values for 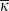 obtained numerically. The numerical values for 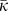 were obtained using fourth-order Runge-Kutta integration of the evolutionary dynamics equations. For 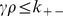, we used the initial condition 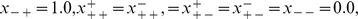 while for 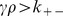 we used the initial condition 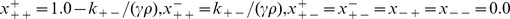. We iterated for 100,000 time steps of size 0.001.

In order to analyze the effect of the conjugation rate 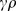 on 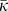 we took the first derivative with respect to 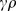, and observed that 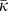 is a decreasing function of 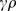, going from 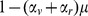 for 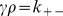 to 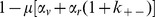 as 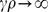 (Figure 3).

**Figure 4:**
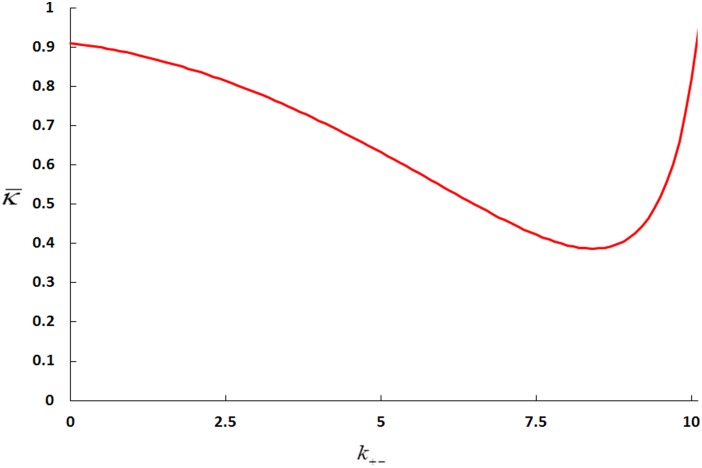
A graphical solution for the population mean fitness _
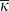
_ versus 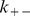 (the first order rate constant corresponding to the transition from the de-repressed to the repressed state of the conjugators). The values of 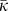 were calculated from Eq. (12) with the parameter values: 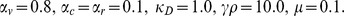

We proceeded with a similar analysis for the repression rate 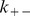 and calculated the first and second derivatives of the population mean fitness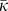 with respect to 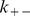. We found that 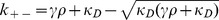 is a local minimum of 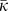 (Figure 4). We can then take the limit of 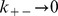, and find that 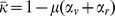 and that 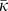 is a decreasing function for 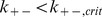. We treat the limit of 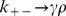 of 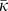 similarly and find that at the range of 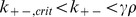,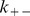 reaches the exact same value of 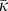 as in the case of 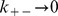 (Figure 4).

## Discussion and Conclusions

**Figure 5:**
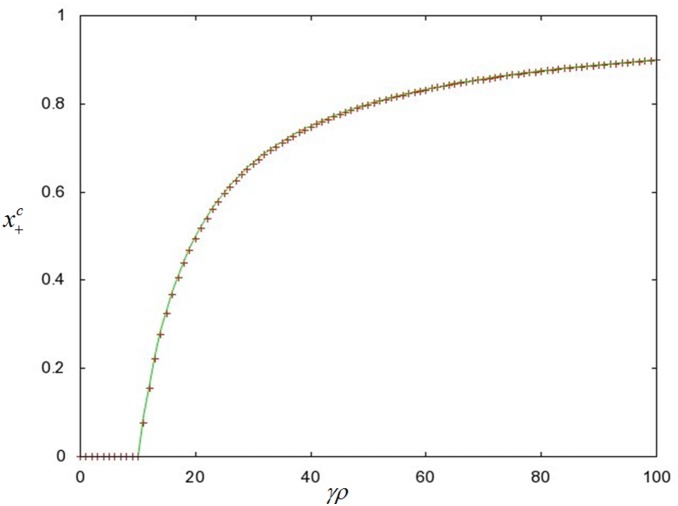
A plot of population fraction of conjugators 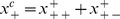 versus the conjugation reaction parameter 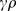. Parameter values are the same as those for figure 3, including the parameters for the Runge-Kutta integration. The solid line represents the zeroth-order analytical expression for 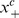, while the pluses represent the values obtained numerically.

**Figure 6:**
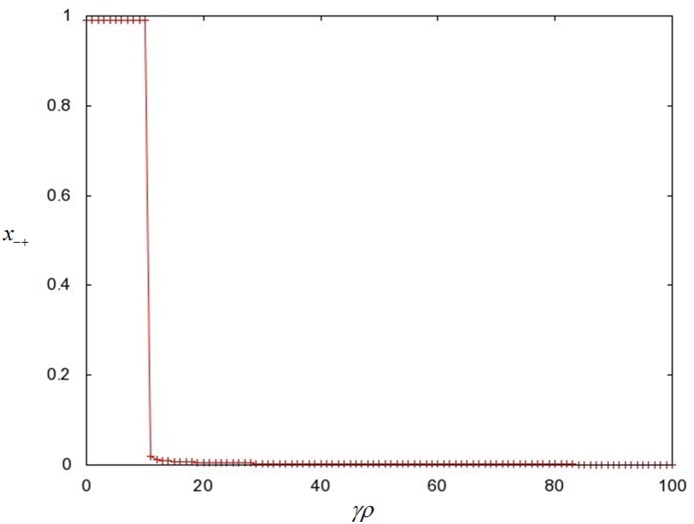
A plot of the population fraction of the non conjugating, antibiotic resistant bacteria sub-population 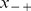 versus the conjugation reaction parameter. The parameter values are the same as those for figure 3, including the parameters for the Runge-Kutta integration.

We have solved our model for the limiting case of weak de-repression 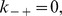 and found a phase transition between a non-conjugator and a conjugator regime (Figures 3, 5, and 6). In our previous work [36], we found a similar phase transition in a system, where all of its population was de-repressed. Such a system, can also be considered as the strong de-repression limit for our current model (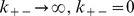). Nevertheless, a phase transition that is related to the conjugation rate in both cases was derived independently for each one of them. Therefore, we conjecture that such a phase transition is ubiquitous whenever the rate of repression is smaller than the rate of conjugation (i.e. 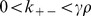).

At first glance, the phase transition that we found for our current model may seem somewhat counter-intuitive, since we would expect no conjugators for all values of 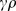 due to the effect of repression. Nevertheless, we can explain the appearance of conjugators from the transient de-repression of the “−” cell upon conjugation. When we examine the trends of the population fractions given in the model with respect to this transition, we observe that when 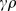 is sufficiently large compared to the characteristic rate of repression, the transient de-repression results in a chain reaction of conjugation-induced de-repressions throughout the population, leading to a stable, and positive, population fraction of conjugators. In other words, once the conjugation rate 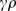 crosses a certain threshold, the transient production of conjugators becomes sufficiently large enough to make the steady-state with no conjugators an unstable one. Any perturbation away from this steady-state takes the system to a steady-state where conjugators are present in the population. Interestingly, we noted from Eq. (14) that the threshold value for 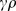 (the rate of conjugation) increases with 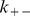 (the rate of repression) and decreases with 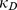 (the antibiotics-induced death rate), sub-section E in the Materials and Methods). This is consistent with the idea, that in order to establish a stable conjugators population, the rate of conjugation has to be slower than the rate of death caused by the antibiotics, as to allow the removal of the non conjugators population via mutation (due to signal fitness peak landscape, this would effectively lead to the removal of the non conjugators) while at the same time, the rate of conjugation has to be significant enough to avoid its effective cessation by repression.

When we introduced bacterial conjugation into our population, the mean fitness of the population in our model has decreased. This is in general agreement with previously published experimental results [43], which attribute this fitness cost to its maintenance by the bacteria. A similar effect was observed in our previous work on the subject [36], although in that specific case the mean fitness has decreased to a minimum for a critical value of the conjugation rate 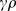, and asymptotically increased to the value of 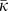 as 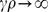 (with respect to its value for 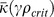).

However, in our current model we have found that, the mean fitness decreases monotonically from 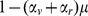 to 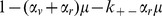 without any observed increase of the mean fitness at the limit of 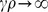. We believe that this may imply on an HGT- driven phase transition that resembles the error-catastrophe phenomenon in viruses that could lead to a critical decrease of the population’s mean fitness [44]. However, the scope of such a phenomenon needs to be further investigated in further theoretical as well as experimental studies.

Furthermore, we noticed that the difference in the values of 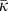
increases with the value of the repression rate 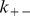. This dependency, implies on the complicated relationship among the parameters 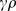, 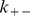 and 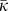
which we have further investigated by differentiating 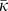 with respect to 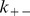 as well as by 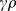. From these derivations we have found that as the conjugation rate is increased, the value of the mean fitness decreases. However, this decrease can be effectively nullified if the rate of repression is high enough. This is the result of a local minimum for 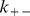 (the repression rate) that occurs at a critical value, which we obtained from differentiating Eq. (12) with respect to 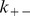 (the derivation of this value can be found in sub-section F in the Materials and Methods and is illustrated by the graphical solution of 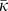
as a function of 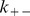 in Figure 4). For 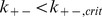 the value of γρ is big enough to lower the population mean fitness while for 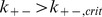 the rate of transition from the de-repressed state to the repressed state is sufficiently large to decrease the difference in the mean fitness caused directly by conjugation. We should note that both the 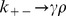 and 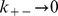 limits of 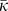 give the same value of 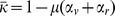. Therefore, once the repression rate is greater than the value of 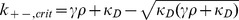, the decrease to the mean fitness caused by conjugation is mitigated to the point that it is effectively cancelled by repression.

Our study illustrates the critical role that repression is likely to have on regulating the process of conjugation and how it can negate the deleterious effect on the fitness of a population. Therefore, while conjugation is disadvantageous in a static environment, its negative effect on the fitness of a population is likely to become negligible if that population can sufficiently repress the plasmid and consequently, restore itself to its original fitness. This would suggest that the role of the bacteria in the actual host-parasite dynamics between the bacteria and the plasmid are much more complicated. While it is known that bacteria can obtain various advantageous traits from plasmids (including antibiotic resistance [3], degradation of unusual substances such as xylene, toluene, napthtalene and phenanthrene [45], resistance to heavy metals [46], [47], and translation of Colicins [48]), they are also required to pay a fitness cost for hosting the plasmid in a static landscape. Previously, it was suggested that repression could reduce the energy costs for the host bacteria [49]. However, experimental results suggested that the amelioration of the fitness cost due to plasmid hosting was possible through evolution of a bacterial population [50]. Based on our results, we propose an alternative mechanism that allows bacteria to ameliorate this fitness cost by repressing their propensity towards conjugation and not only by vertical evolution. Interestingly, this strategy is likely to allow bacterial populations a significantly faster restoration pathway for their fitness loss (due to the maintenance costs of antibiotic drug resistance) then vertical gene transfer (i.e. evolution).

Our current model bears similarities to the Susceptible-Infected-Susceptible (SIS) [51]–[53] class of infectious disease models, where the “−” cells play the role of the Susceptibles (S) and the “+” cells play the role of the Infected (I). If the contact frequency between the S and the I population classes is sufficiently low, so that the I’s revert back to the S state before having an opportunity to infect at least one additional S, then the fraction of I’s in the population drops to a negligible fraction of the population. Above a critical contact frequency, however, the fraction of I’s rises to a finite positive fraction of the population. Our model is in broad agreement with the behavior of actual conjugative plasmid systems, where it was observed that conjugative plasmids can spread in an infectious manner above a critical population density [38], [54], [55]. Typically this decrease in the population mean fitness is explained by the presence of Male Specific Phages (MSPs) [56], but in our model this factor is not accounted for, resulting in a decrease of the mean fitness that is a direct result of mutations that the plasmids go through when being replicated within the host bacteria.

We would like to also note recent studies that have suggested bacterial conjugation as a platform for the development of conjugation-based antibiotics [57]. In light of these studies, our results may have implications about the pharmaceutical limits that need to be considered for such antibiotic agents.

Finally, we believe that developing mathematical models that can correlate known data is important, as it indicates that the basic mathematical framework is reasonable, and may therefore serve as a foundation for building more sophisticated quantitative and predictive models. For future work, we will need to take into account more biological features of conjugation, such as regulation of plasmid copy number, MSPs and plasmid compatibility classes. Furthermore, we will need to move away from static landscapes and consider the role that conjugation has in allowing a population to adapt to new environments.

## Materials and Methods

### A. Proof That There Are No Non-resistant Organisms for 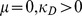


If we define 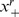
to be the total fraction of resistant organisms, and 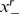
to be the total fraction of non-resistant organisms, then we have 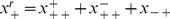 and 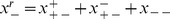. We then have that 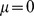,
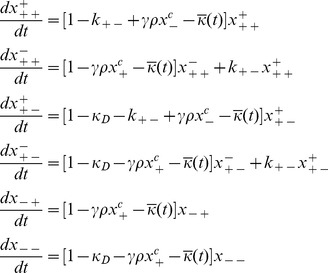
(8)


If we assume that the system converges to a stable steady-state, then we must have that 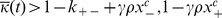. If, for 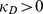 there are non-resistant organisms in the population, then either 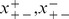 or 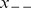 are strictly positive. If 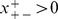, then we have 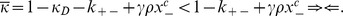 Therefore, 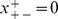, so either 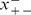 or 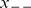
are >0, which gives 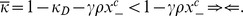
Thus, there are no non-resistant organisms in the population at steady-state for µ = 0.

### B. Proof That 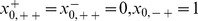 for 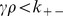


Let us suppose that 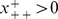 for some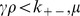. Then the first line in Eq. (3) gives 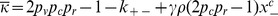. Now, assuming that our system converges to a stable steady-state, we also have from the the fifth line in Eq. (3) that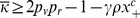, and so 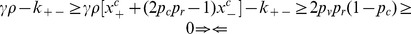. Therefore, 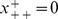 for 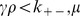, which implies that 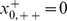.

Suppose then that, 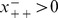
for 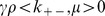. Then from Eq. (3) we have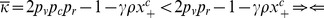, hence 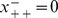 for 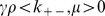.

Taking the limit as 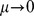, we obtain 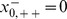
for 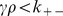. Now, when 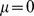, we have 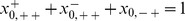, and so for we obtain 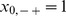.

### C. Proof That 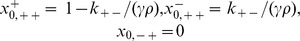 for 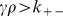


Given some 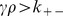, suppose that 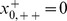. Then since that are no non-resistant organisms for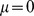, we have that 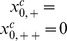 and 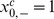. This implies that
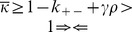
, and so we must have that 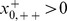. This gives, 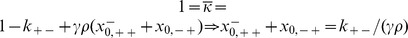, and so 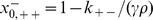
_._

Now, the fifth line of Eq. (3) gives,
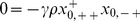
(9)


from which it follows that 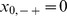, and so 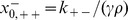.

### D. Derivation of the Small-µ Expressions for 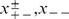


For small µ, we may write the first-order expressions for 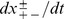 and 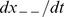. We obtain,
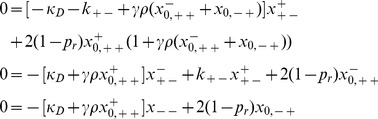
(10)


For 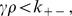
we have 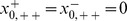,and 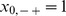, which implies 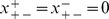, and.


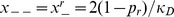
, thus 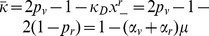.

For 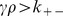
we have 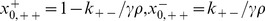, and 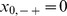. This gives,
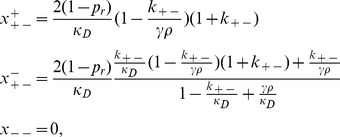
(11)


and so the mean fitness at steady-state is given by,
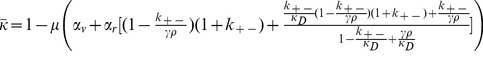
(12)


### E. Derivation of 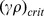 for 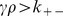


In order to find the critical value of 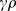, we compute the derivative of Eq. (12) with respect to
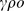
and derive the following expression:
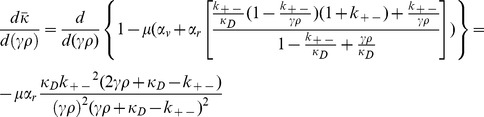
(13)


Equating this term to zero allows us to find 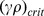:
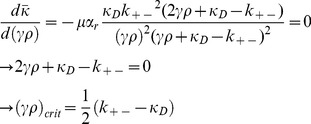
(14)


### F. Derivation of 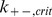


In order to find the critical value of 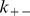
we take the derivative of Eq. (12) with respect to 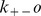
and get the following expression:
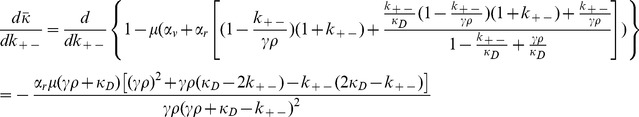
(15)


By equating this expression to zero we get the following quadratic equation for 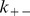:
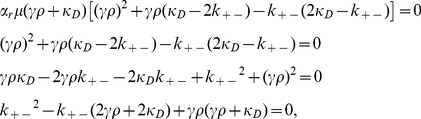
(16)


which allows us to find the values for 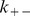 that solve the aforementioned equation:
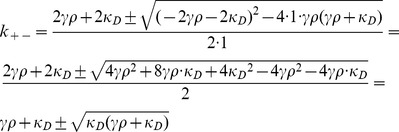
(17)


Once we take into account the singularity of 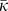 at 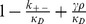 we are left only with the following:
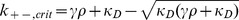
(18)


Note that one can compute that.
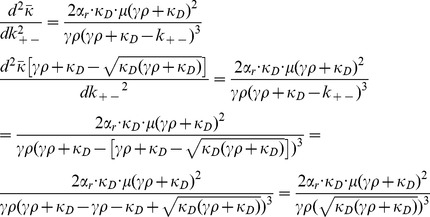
(19)


If we take into account the positivity of 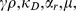 we see that 
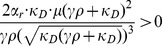
, and so the critical value 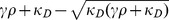
is indeed a (local) minimum value of 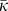.
