# Correction: Repression/Depression of Conjugative Plasmids and Their Influence on the Mutation-Selection Balance in Static Environments

**DOI:** 10.1371/journal.pone.0099919

**Published:** 2014-06-04

**Authors:** The PLOS ONE Staff

## Notice of Republication

This article was republished on May 20th, 2014, due to Figure 4, Figure 5, and Figure 6 being ordered incorrectly. Please download the PDF again to view the corrected article. The originally published, uncorrected article and the republished, corrected article are provided here for reference.

## Supporting Information

File S1 **Originally published, uncorrected article.**
(PDF)

File S2 **Republished, corrected article.**
(PDF)
