# Correction: Repression/Depression of Conjugative Plasmids and Their Influence on the Mutation-Selection Balance in Static Environments

**DOI:** 10.1371/journal.pone.0112353

**Published:** 2014-10-27

**Authors:** The PLOS ONE Staff

The first author’s name is spelled incorrectly. The correct name is: Yoav Atsmon-Raz. The correct citation is: Atsmon-Raz Y, Tannenbaum ED (2014) Repression/Depression of Conjugative Plasmids and Their Influence on the Mutation-Selection Balance in Static Environments. PLoS ONE 9(5): e96839. doi:10.1371/journal.pone.0096839
