# Supplementary material for: Correction: Repression/Depression of Conjugative Plasmids and Their Influence on the Mutation-Selection Balance in Static Environments
Source: PLoS One. 2014 Jun 4;9(6):e99919. doi: 10.1371/journal.pone.0099919 (PMC4045931; doi:10.1371/journal.pone.0099919)
Supplement: File S2 — Republished, corrected article. (PDF) [file pone.0099919.s002.pdf]

Repression/Depression of Conjugative Plasmids and Their Influence on the Mutation-Selection Balance in Static Environments

Yoav Raz*, Emmanuel David Tannenbaum

Department of Chemistry, Ben-Gurion University of the Negev, Beér-Sheva, Israel

Abstract

We study the effect that conjugation-mediated Horizontal Gene Transfer (HGT) has on the mutation-selection balance of a population in a static environment. We consider a model whereby a population of unicellular organisms, capable of conjugation, comes to mutation-selection balance in the presence of an antibiotic, which induces a first-order death rate constant κ_D for genomes that are not resistant. We explicitly take into consideration the repression/de-repression dynamics of the conjugative plasmid, and assume that a de-repressed plasmid remains temporarily de-repressed after copying itself into another cell. We assume that both repression and de-repression are characterized by first-order rate constants k_{+-} and k_{-+}, respectively. We find that conjugation has a deleterious effect on the mean fitness of the population, suggesting that HGT does not provide a selective advantage in a static environment, but is rather only useful for adapting to new environments. This effect can be ameliorated by repression, suggesting that while HGT is not necessarily advantageous for a population in a static environment, its deleterious effect on the mean fitness can be negated via repression. Therefore, it is likely that HGT is much more advantageous in a dynamic landscape. Furthermore, in the limiting case of a vanishing spontaneous de-repression rate constant, we find that the fraction of conjugators in the population undergoes a phase transition as a function of population density. Below a critical population density, the fraction of conjugators is zero, while above this critical population density the fraction of conjugators rises continuously to one. Our model for conjugation-mediated HGT is related to models of infectious disease dynamics, where the conjugators play the role of the infected (I) class, and the non-conjugators play the role of the susceptible (S) class.

Citation: Raz Y, Tannenbaum ED (2014) Repression/Depression of Conjugative Plasmids and Their Influence on the Mutation-Selection Balance in Static Environments. PLoS ONE 9(5): e96839. doi:10.1371/journal.pone.0096839

Editor: Eric Cascales, Centre National de la Recherche Scientifique, Aix-Marseille Université, France

Received July 1, 2011; **Accepted** April 13, 2014; **Published** May 8, 2014

Copyright: © 2014 Raz, Tannenbaum. This is an open-access article distributed under the terms of the Creative Commons Attribution License, which permits unrestricted use, distribution, and reproduction in any medium, provided the original author and source are credited.

Funding: This research was supported by the U.S.-Israel Binational Science Foundation (www.bsf.org.il) and by the Israel Science Foundation (www.isf.org.il). The funders had no role in study design, data collection and analysis, decision to publish, or preparation of the manuscript.

Competing Interests: The authors have declared that no competing interests exist.

* E-mail: razyoav@post.bgu.ac.il

Introduction

Horizontal Gene Transfer (HGT) is defined as the transfer or exchange of genetic information between two organisms, that does not involve the transmission of genetic information from parent to daughter as a result of replication, a process known as *vertical transmission of genetic information* [1,2]. Since it was initially characterized in the 1940s [3], it has gradually become evident that HGT is one of the major sources of genomic change in prokaryotes [2]. As a result of its recognized importance for shaping the genomes of prokaryotes, a complete theory describing the evolutionary dynamics of prokaryotes must take HGT into account [2]. Further, the fact that HGT plays a major role in shaping prokaryotic genomes means that HGT must be considered when reconstructing phylogenetic trees [1], which of course complicates any effort to understand the evolutionary relationships amongst bacterial strains, and to trace the evolutionary histories of various genes. The existence of HGT also has consequences for public health, since HGT is believed to be primarily responsible for the rapid spread of antibiotic drug resistance in bacterial populations [2,4–8].

Despite the large influence that HGT has on the evolutionary dynamics of prokaryotic populations [2], with a few notable exceptions [9–12], there has been relatively little theoretical work attempting to incorporate HGT into evolutionary models. Indeed, it is safe to say that the bulk of the work in evolutionary biology has focused on point-mutations as the primary source of genetic variation [13]. There are two main reasons for this: First of all, the simplest evolutionary models are the ones that consider point-mutations as the only source of genetic variation. Yet even these models can exhibit fairly non-trivial behavior, and there is a wide range of additional effects that may be considered within the point-mutation framework (e.g., conservative versus semiconservative replication, fitness landscapes, polysomic genomes, genetic instability, genetic repair and mutators, finite-size effects [9,13–21]). Thus, already at the simplest level of point-mutations, there is a fairly large class of evolutionary models that may be analyzed. Second, for quite some time in biology it was believed that point-mutations are indeed the primary source of genetic change in organisms. This assumption underlies the concept of the so-called "tree of life" that was used to organize phylogenetic data. With the realization of the importance of HGT, there have been suggestions to replace the tree analogy with that of a web or a ring [1,22–25].

Bacterial genomes typically consist of a single, large, circular chromosome, in addition to several smaller, circular chromosomes known as plasmids. Bacterial plasmids can move between bacteria, often between bacteria of different strains, via a process known as *conjugation*.[26–29]. If these plasmids contain genes for an adaptive trait such as antibiotic drug resistance, then conjugation can significantly speed up the rate at which such an adaptive trait spreads throughout a population.[30–33]. Conjugation is believed to be one of the most important forms of HGT, so that both phenomenological and quantitative models of processes such as the emergence of antibiotic drug resistance will need to properly account for this behavior.

The best characterized bacterial conjugation system is the F^+/F^- system [28]. Here, a bacterium containing what is termed an F-plasmid fuses with a bacterium lacking the F-plasmid. The bacterium containing the F-plasmid is termed an F^+ bacterium, while the bacterium that does not contain this plasmid is termed an F^- bacterium. When an F^+ bacterium meets an F^- bacterium, it transfers one of the strands of the F-plasmid to the F^- bacterium via a pilus. Once a strand of the s F-plasmid has been transferred from the F^+ bacterium to the F^- bacterium, a copy of the plasmid in both cells is produced by daughter strand synthesis using the DNA template strands. The F^- bacterium then becomes an F^+ bacterium that transcribes its own pilus, and is able to transfer the F^-plasmid to other bacteria in the population (Figure 1).

The F^+/F^- system is in some ways atypical for bacterial conjugation systems, due to its conjugative machinery being chronically in a de-repressed state, while in nature most conjugative plasmid systems are repressed via the expression of T4SS modules [34,35]. Nevertheless, the F-plasmid system is still one of the best characterized bacterial conjugation systems, and it can be considered as broadly representative of all known bacterial conjugation systems. Therefore, it makes sense to base mathematical models of conjugation-mediated HGT on the F-plasmid system.

In recent work [36], the authors developed mathematical models describing the role that conjugation has on the mean fitness of unicellular populations in static environments, and found that conjugation-mediated HGT does not confer a selective advantage to the bacterial population (indeed conjugation-mediated HGT was found to have a slightly deleterious effect on the mean fitness of a population). However, in this work, it was assumed that the conjugation machinery is permanently de-repressed. Here, we extend our previous work by introducing the repression/derepression mechanism into our original model [13] of a bacterial population that transfers genetic information among its members both vertically and horizontally (Figure 2).

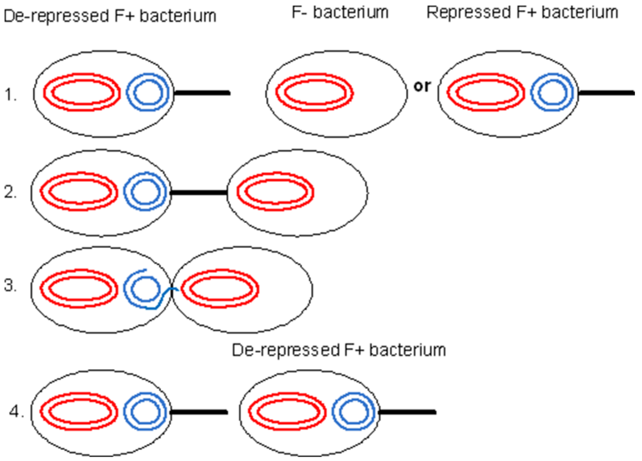

Figure 1. The conjugation process for F+ bacterium with F- bacterium as detailed in our model: 1. A de-repressed bacterium comes within proximity of an F- bacterium/repressed F+ bacterium. 2. The F+ bacterium's pili interacts with the F- bacterium's membrane. 3. The F+ plasmid releases a strand of its DNA to the F- bacterium via conjugation machinery. 4. A new de-repressed F+ bacterium is formed.
doi:10.1371/journal.pone.0096839.g001

Results

1. Definition of the Model

Our model assumes a unicellular population of asexually reproducing bacteria, where the bacterial genome consists of two chromosomes. The first chromosome is the main circular bacterial chromosome that transfers its genetic information via vertical transmission (i.e. replication from one generation to the next). The second chromosome is taken to be the much smaller conjugative plasmid, which is assumed to consist of two regions: (1) A "conjugation" region, comprising the various genes necessary for bacterial conjugation. (2) An "antibiotic resistance" region, comprising the genes necessary for conferring resistance to a given antibiotic. Since each chromosome is assumed to be a double-stranded, semiconservatively replicating DNA molecule, we define probabilities for the error-free replication of a template strand from each of the various regions of the bacterial genome. To this end, we let p_v denote the probability that a template strand from the main bacterial chromosome produces a daughter chromosome without introducing any new point-mutations. We define p_c and p_r analogously for the portions of the bacterial plasmid coding for conjugation and antibiotic drug resistance. We assume that there are master sequences for the main bacterial genome, the conjugation and antibiotic drug resistance regions of the plasmid. We assume that any mutation to these master sequences renders the sequences non-functional. We further assume that the bacterial genome controls viability, and so a bacterium with a master, or wild-type, copy of its main chromosome, has a normalized first-order growth rate constant of 1, while a bacterium with a mutated main chromosome has a first-order growth rate constant of 0. The assumption that a single mutation renders the master sequence non-functional is known as the single-fitness-peak approximation [13]. Although it is an oversimplification of the true dependence of functionality on the gene sequence, it nevertheless captures the fact that only a small fraction of sequences are likely to yield a gene that encodes the desired function. As a result, this approximation has been known to give quantitative results in certain cases [37].

Given its simplicity, it therefore makes sense to start with this assumption before moving on to more complicated fitness models. We assume that a viable bacterium whose resistance region of the plasmid corresponds to the wild-type is unaffected by the presence of an antibiotic, while a viable bacterium with a mutated portion of this resistance region is killed by the antibiotic at a rate characterized by a first-order rate constant κ_D.

Bacterial conjugation is modeled as a second-order rate process characterized by a rate constant γ. We also assume that, as the size of the bacterial population changes, the system volume changes so as to maintain a constant population density ρ while assuming that ρ remains constant. It should be noted that in this context, the only bacteria whose population we are tracking are

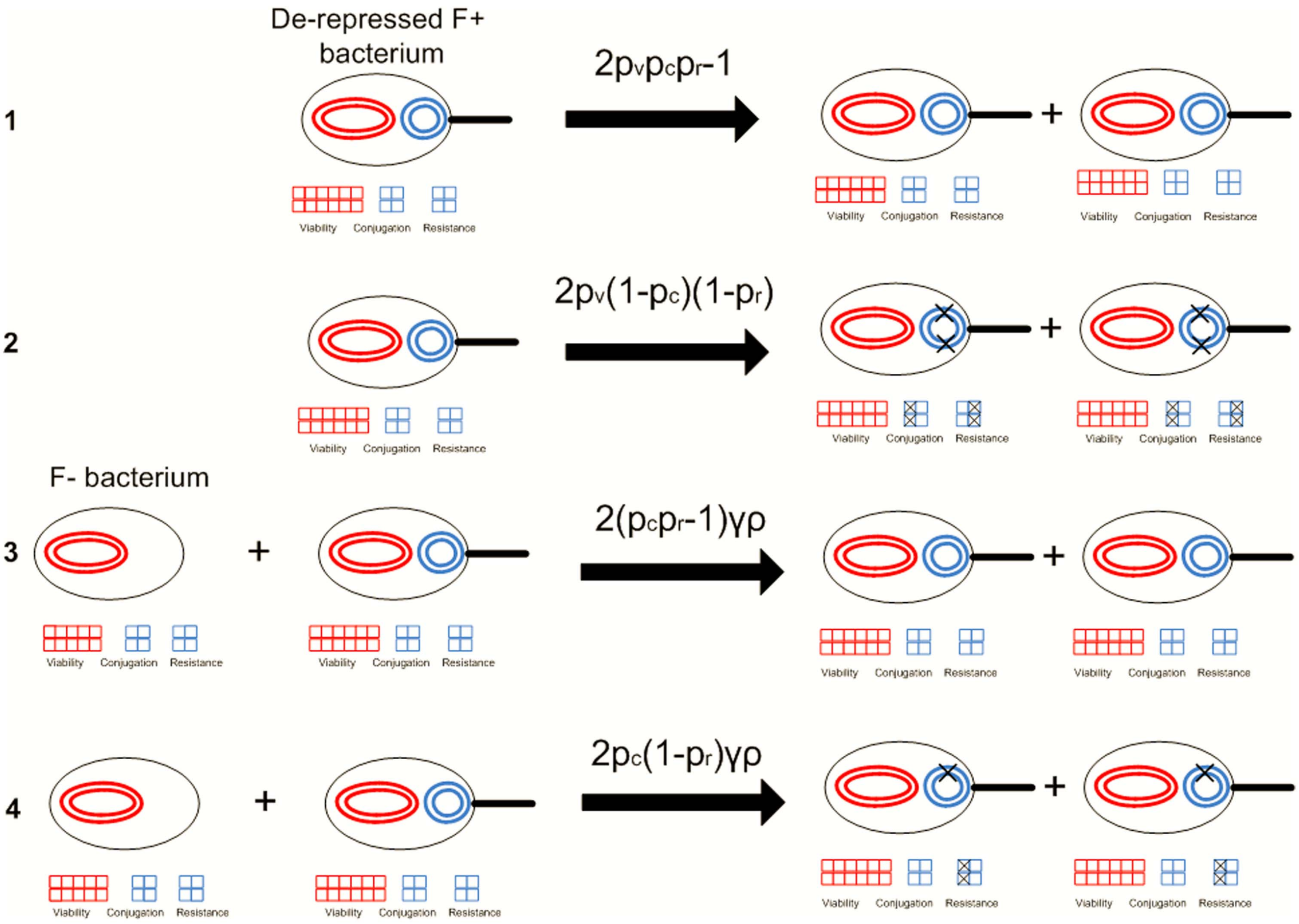

Figure 2. Examples for vertical replication and horizontal gene transfer within the framework of our model and the consequential propogation of mutations throughout the population: 1. Mutation-free vertical replication of the de-repressed, fully viable subpopulation that is notated as x^{+}_{++}. 2. Vertical replication followed by a mutation to the conjugation and antibiotic resistance genes in the plasmid. 3. Mutation-free bacterial conjugation between an F+ and an F- bacterium that leads to the formation of two antibiotic resistant, conjugation capable, derepressed bacteria. 4. Horizontal transfer between an F+ and F- bacterium followed by a mutation to the antibiotic resistance gene of both conjugants, leading to no drug resistance for these bacteria. $p_{v,c,r}$ respectively notate the probabilities for the bacterium replicating its viability, conjugation, and resistance portions of the genome in an error-free manner while $\gamma\rho$ is the second order reaction parameter of the conjugation rate.
doi:10.1371/journal.pone.0096839.g002

the viable ones. The idea behind this assumption is that the bacteria each take up a certain amount of space, and so, as the bacterial population grows, the total system volume must increase as well. Bacterial conjugation can only occur between a viable bacterium whose conjugation genes are de-repressed, and a viable bacterium whose conjugation genes are repressed. The repression/de-repression dynamics is assumed to be characterized by first-order rate constants $k_{+-}$and k_{-+}, respectively, corresponding to transitions from the de-repressed to the repressed state, and from the repressed state to the de-repressed state, respectively. However, for the purposes of this paper, we will assume that $k_{-+}=0$, corresponding to the limit of weak de-repression, which is believed to characterize the repression/de-repression dynamics of actual systems [34,38].

During bacterial conjugation, we assume that a template strand of the plasmid from the de-repressed bacterium, which is labelled the "+" bacterium, is transferred to the repressed, or "−", bacterium, and that daughter strand synthesis occurs in both bacteria to reconstitute the plasmids. We assume that the plasmid transferred from the "+" bacterium replaces the plasmid in the "−" bacterium, and inherits the de-repressed state of the parent plasmid. The assumption of plasmid replacement is a simplification that will need to be re-examined in future research, where we anticipate developing more accurate models that allow for variable plasmid numbers in the bacterial cell. The basis for this assumption derives from the observation that plasmids of similar compatibility classes cannot co-exist in the same cell [39,40], and that bacteria can control the number of plasmids in the cell [40–42]. While these observations do not directly translate into a plasmid replacement model, we believe that they do provide some justification for this assumption.

Finally, we assume that replication errors are due to mismatches during daughter strand synthesis that are sub-sequentially fixed in the genome. We let ε denote the mismatch probability per base-pair, so that ε/2 is the probability of making a mismatch, which is fixed as a mutation in the genome. If L_v,L_c,L_r denote the lengths of the viability, conjugation, and resistance portions of the genome, respectively, then we have that $p_v=(1-\varepsilon/2)^{L_v}$, $p_c=(1-\varepsilon/2)^{L_c}$ and $p_r=(1-\varepsilon/2)^{L_r}$.

Now, following the standard procedure in population genetics and quasispecies models [13,19], we may allow the genome length $L = L_v + L_c + L_r$ to become very large while holding $\mu = \varepsilon L$ constant. Defining $\alpha_v = L_v/L, \alpha_c = L_c/L, \alpha_r = L_r/L$, we then obtain,

$$p_{v,c,r} = e^{-\alpha_{v,c,r}\mu/2} \tag{1}$$

2. Evolutionary Dynamics Equations

Here, we define $x^{\circ}_{\Delta\Box}$ to be the fraction of the population consisting of viable bacteria such that the conjugator region is of type $\triangle$, and the resistance region is of type $\Box$, where a type of "+" means that the given region is of the wild-type, while a type of "−" means that the given region differs from the wild-type. When $\triangle = +$, corresponding to a conjugator, then $^{\circ} = \pm$, corresponding to the genes for conjugation being de-repressed ($^{\circ} = +$) or repressed ($^{\circ} = -$). Since we are only tracking viable bacteria, we should note that $x^+_{++} + x^-_{++} + x^+_{+-} + x^-_{+-} + x_{-+} + x_{--} = x$.

We define x^c_+ to be the total fraction of viable bacteria that are conjugators, so that $x^c_+ = x^+_{++} + x^+_{+-}$, and we define x^c_- to be the total fraction of viable bacteria that are non-conjugators, so that $x^c_- = x^-_{++} + x^-_{+-} + x_{-+} + x_{--}$. Therefore, allowing us to summarize all of the processes that are relevant for each subpopulation in our model into a generalized list of balance equations which are described by Eq. (2):

error-free semi-conservative replication (vertical gene transfer) :

$$x^{\pm}_{\pm\pm} \xrightarrow{2p_v p_j p_k - 1} x^{+}_{\pm\pm} + x^{+}_{\pm\pm}$$

non-plasmid-restricted mutation (as a result of vertical gene transfer) :

$$x^{\pm}_{\pm\pm} \xrightarrow{2p_v p_j p_k} x^{+*}_{\pm\pm} + x^{+*}_{\pm\pm}$$

error-free bacterial conjugation (horizontal gene trasnfer) :

$$x^c_+ + x^c_- \xrightarrow{\gamma\rho(2p_j p_k - 1)} x^c_+ + x^c_+ \tag{2}$$

plasmid-restricted mutation (as a result of bacterial conjugation) :

$$x^c_+ + x^c_- \xrightarrow{\gamma\rho 2 p_j p_k} x^c_- + x^c_-$$

repression :

$$x^{+}_{\pm\pm} \xrightarrow{k_{+-}} x^{-}_{\pm\pm}$$

death by antibiotics :

$$x^{\pm}_{\pm -} \xrightarrow{\kappa_D} \text{dead bacterium}$$

We note that p_j denotes two possible values for the conjugation gene - p_c when the gene is copied or conjugated in an error-free manner, or $1-p_c$ when the gene is copied incorrectly. In a similar manner, p_k denotes two possible values for the antibiotics resistance gene - p_r or $1-p_r$.

Putting everything together, we obtain that the evolutionary dynamics of the bacterial population is governed by the following system of equations:

$$\begin{aligned}
\frac{dx^+_{++}}{dt} &= [2p_v p_c p_r - 1 - k_{+-} + \gamma\rho(2p_c p_r - 1)x^c_- - \bar{\kappa}(t)]x^+_{++} \\
\frac{dx^-_{++}}{dt} &= [2p_v p_c p_r - 1 - \gamma\rho x^c_+ - \bar{\kappa}(t)]x^-_{++} + k_{+-}x^+_{++} \\
\frac{dx^+_{+-}}{dt} &= [2p_v p_c - 1 - \kappa_D - k_{+-} + \gamma\rho(2p_c - 1)x^c_- - \bar{\kappa}(t)]x^+_{+-} + \\
&\quad 2p_c(1-p_r)x^+_{++}(p_v + \gamma\rho x^c_-) \\
\frac{dx^-_{+-}}{dt} &= [2p_v p_c - 1 - \kappa_D - \gamma\rho x^c_+ - \bar{\kappa}(t)]x^-_{+-} + \\
&\quad k_{+-}x^+_{+-} + 2p_v p_c(1-p_r)x^-_{++} \\
\frac{dx_{-+}}{dt} &= [2p_v p_r - 1 - \gamma\rho x^c_+ - \bar{\kappa}(t)]x_{-+} + \\
&\quad 2p_v(1-p_c)p_r(x^+_{++} + x^-_{++}) \\
&\quad + \gamma\rho[2(1-p_c)p_r]x^+_{++}x^c_- \\
\frac{dx_{--}}{dt} &= [2p_v - 1 - \kappa_D - \gamma\rho x^c_+ - \bar{\kappa}(t)]x_{--} + \\
&\quad 2p_v(1-p_c)(1-p_r)(x^+_{++} + x^-_{++}) \\
&\quad + 2p_v(1-p_c)(x^+_{+-} + x^-_{+-}) + 2p_v(1-p_r)x_{-+} + \\
&\quad \gamma\rho[2(1-p_c)]x^c_-[(1-p_r)x^+_{++} + x^+_{+-}]
\end{aligned} \tag{3}$$

By summarizing all of these equations we get that $\bar{\kappa}(t) = 2p_v - 1 - \kappa_D(x^+_{+-} + x^-_{+-} + x_{--})$ is the mean fitness of the population, or the first-order growth rate constant of the population as a whole. Finally, we'd also like to note that the form of the equations detailed by Eq. (3) is identical to what would be obtained if we made a more realistic assumption that the population was growing in a chemostat. [13].

3. Mutation-selection Balance

We solve our system of equations for the mutation-selection balance for the case when μ is small enough so to allow us to avoid the error-catastrophe region. This allows us to apply a first order taylor expansion to Eq. (1), which gives us simpler terms for p_v,p_c and p_r which are $p_v = 1 - \alpha_v\mu/2; p_c = 1 - \alpha_c\mu/2$ and $p_r = 1 - \alpha_r\mu/2$ (the probabilities for an error-free replication of bacterium's chromosome, conjugation gene and resistance gene respectively). Our first goal is to determine the initial conditions which will allow our system to converge while maintaining physical context. We proceed by considering a preliminary condition for the antibiotics-related deathκ_D which must be greater then the value of zero while denoting $x^+_{0,++}, x^-_{0,++}, x^+_{0,+-}, x^-_{0,+-}, x_{0,-+}, x_{0,--}$ as the $\mu = 0$ values of $x^+_{++}, x^-_{++}, x^+_{+-}, x^-_{+-}, x_{-+}, x_{--}$, respectively. We show in sub-section A from the Materials and Methods that this condition brings forth that there are no non-resistant organisms in the system when $\mu = 0$ so that $x^+_{0,+-} = x^-_{0,+-} = x_{0,--} = 0$. From this point, we separate our analysis into two regions based on the differences between the values of$\gamma\rho$and k_{+-}. The first region obeys the condition of $\gamma\rho < k_{+-}$ and we have proved that for this region $x^+_{0,++} = x^-_{0,++} = 0$and $x_{0,-+} = 1$(sub-section B from the Materials and methods). The second region obeys the condition of $\gamma\rho > k_{+-}$ and we proved that for this region $x^+_{0,++} = 1 - k_{+-}/(\gamma\rho), x^-_{0,++} = k_{+-}/(\gamma\rho), x_{0,-+} = 0$ (sub-section C of the Materials and methods). We applied these to Eq. (3) and

found two separate solutions for $x^{+}_{+-}, x^{-}_{+-}, x_{--}$ and consequently were able to compute the terms for the population mean fitness for each of these regions (sub-section D in Materials and methods). For $\gamma\rho < k_{+-}$, we have $x^{+}_{0,++} = x^{-}_{0,++} = 0$,and $x_{0,-+} = 1$ while $x^{+}_{+-}, x^{-}_{+-}, x_{--}$ have the following values:

$$\begin{aligned} x^{+}_{+-} &= 0 \\ x^{-}_{+-} &= 0 \\ x_{--} &= \frac{2(1-p_r)}{\kappa_D} \end{aligned} \quad (4)$$

Therefore, in selection-mutation balance their population mean fitness is:

$$\bar{\kappa} = 1 - (\alpha_v + \alpha_r)\mu \quad (5)$$

The second solution is for $\gamma\rho > k_{+-}$ when $x^{+}_{0,++} = 1 - k_{+-}/\gamma\rho, x^{-}_{0,++} = k_{+-}/\gamma\rho$ and $x_{0,-+} = 0$ when we obtain,

$$\begin{aligned} x^{+}_{+-} &= \frac{2(1-p_r)}{\kappa_D}\left(1 - \frac{k_{+-}}{\gamma\rho}\right)(1 + k_{+-}) \\ x^{-}_{+-} &= \frac{2(1-p_r)}{\kappa_D}\left[\frac{k_{+-}(\gamma\rho - k_{+-})(1 + k_{+-}) + k_{+-}\kappa_D}{\gamma\rho(\kappa_D + \gamma\rho - k_{+-})}\right] \\ x_{--} &= 0 \end{aligned} \quad (6)$$

from which we derive the selection-mutation balance mean fitness of:

$$\bar{\kappa} = 1 - \mu\left(\alpha_v + \alpha_r\left[\left(1 - \frac{k_{+-}}{\gamma\rho}\right)(1 + k_{+-}) + \frac{\frac{k_{+-}}{\kappa_D}\left(1 - \frac{k_{+-}}{\gamma\rho}\right)(1 + k_{+-}) + \frac{k_{+-}}{\gamma\rho}}{1 - \frac{k_{+-}}{\kappa_D} + \frac{\gamma\rho}{\kappa_D}}\right]\right) \quad (7)$$

In order to analyze the effect of the conjugation rate $\gamma\rho$ on $\bar{\kappa}$ we took the first derivative with respect to $\gamma\rho$, and observed that $\bar{\kappa}$ is a decreasing function of $\gamma\rho$, going from $1-(\alpha_v+\alpha_r)\mu$ for $\gamma\rho = k_{+-}$ to $1-\mu[\alpha_v+\alpha_r(1+k_{+-})]$ as $\gamma\rho \to \infty$ (Figure 3).

We proceeded with a similar analysis for the repression rate k_{+-} and calculated the first and second derivatives of the population mean fitness$\bar{\kappa}$ with respect to k_{+-}. We found that $k_{+-} = \gamma\rho + \kappa_D - \sqrt{\kappa_D(\gamma\rho + \kappa_D)}$ is a local minimum of $\bar{\kappa}$ (Figure 4). We can then take the limit of $k_{+-} \to 0$, and find that $\bar{\kappa} = 1 - \mu(\alpha_v + \alpha_r)$ and that $\bar{\kappa}$ is a decreasing function for $k_{+-} < k_{+-,crit}$. We treat the limit of $k_{+-} \to \gamma\rho$ of $\bar{\kappa}$ similarly and find that at the range of $k_{+-,crit} < k_{+-} < \gamma\rho, k_{+-}$ reaches the exact same value of $\bar{\kappa}$ as in the case of $k_{+-} \to 0$ (Figure 4).

Discussion and Conclusions

We have solved our model for the limiting case of weak de-repression $k_{-+} = 0$, and found a phase transition between a non-conjugator and a conjugator regime (Figures 3, 5, and 6). In our previous work [36], we found a similar phase transition in a system, where all of its population was de-repressed. Such a system, can also be considered as the strong de-repression limit for our current model ($k_{+-} \to \infty$, $k_{+-} = 0$). Nevertheless, a phase transition that is related to the conjugation rate in both cases was derived independently for each one of them. Therefore, we conjecture that such a phase transition is ubiquitous whenever the rate of repression is smaller than the rate of conjugation (i.e. $0 < k_{+-} < \gamma\rho$).

At first glance, the phase transition that we found for our current model may seem somewhat counter-intuitive, since we would expect no conjugators for all values of $\gamma\rho$ due to the effect of repression. Nevertheless, we can explain the appearance of conjugators from the transient de-repression of the "$-$" cell upon conjugation. When we examine the trends of the population fractions given in the model with respect to this transition, we observe that when $\gamma\rho$ is sufficiently large compared to the characteristic rate of repression, the transient de-repression results in a chain reaction of conjugation-induced de-repressions throughout the population, leading to a stable, and positive, population fraction of conjugators. In other words, once the conjugation rate $\gamma\rho$ crosses a certain threshold, the transient production of conjugators becomes sufficiently large enough to make the steady-state with no conjugators an unstable one. Any perturbation away from this steady-state takes the system to a steady-state where conjugators are present in the population. Interestingly, we noted from Eq. (14) that the threshold value for $\gamma\rho$ (the rate of conjugation) increases with k_{+-} (the rate of repression) and decreases with κ_D (the antibiotics-induced death rate), sub-section E in the Materials and Methods). This is consistent with the idea, that in order to establish a stable conjugators population, the rate of conjugation has to be slower than the rate of death caused by the antibiotics, as to allow the removal of the non conjugators population via mutation (due to signal fitness peak landscape, this would effectively lead to the removal of the non conjugators) while at the same time, the rate of conjugation has to be significant enough to avoid its effective cessation by repression.

When we introduced bacterial conjugation into our population, the mean fitness of the population in our model has decreased. This is in general agreement with previously published experimental results [43], which attribute this fitness cost to its maintenance by the bacteria. A similar effect was observed in our previous work on the subject [36], although in that specific case the mean fitness has decreased to a minimum for a critical value of the conjugation rate $\gamma\rho$, and asymptotically increased to the value of $\bar{\kappa}$ as $\gamma\rho \to \infty$ (with respect to its value for $\bar{\kappa}(\gamma\rho_{crit})$).

However, in our current model we have found that, the mean fitness decreases monotonically from $1-(\alpha_v+\alpha_r)\mu$ to $1-(\alpha_v+\alpha_r)\mu - k_{+-}\alpha_r\mu$ without any observed increase of the mean fitness at the limit of $\gamma\rho \to \infty$. We believe that this may imply on an HGT- driven phase transition that resembles the error-catastrophe phenomenon in viruses that could lead to a critical decrease of the population's mean fitness [44]. However, the scope of such a phenomenon needs to be further investigated in further theoretical as well as experimental studies.

Furthermore, we noticed that the difference in the values of $\bar{\kappa}$increases with the value of the repression rate k_{+-}. This dependency, implies on the complicated relationship among the parameters $\gamma\rho$, k_{+-} and $\bar{\kappa}$which we have further investigated by differentiating $\bar{\kappa}$ with respect to k_{+-} as well as by $\gamma\rho$. From these derivations we have found that as the conjugation rate is increased, the value of the mean fitness decreases. However, this decrease can be effectively nullified if the rate of repression is high enough. This is the result of a local minimum for k_{+-} (the repression rate) that occurs at a critical value, which we obtained from differentiating Eq. (12) with respect to k_{+-} (the derivation of this value can be

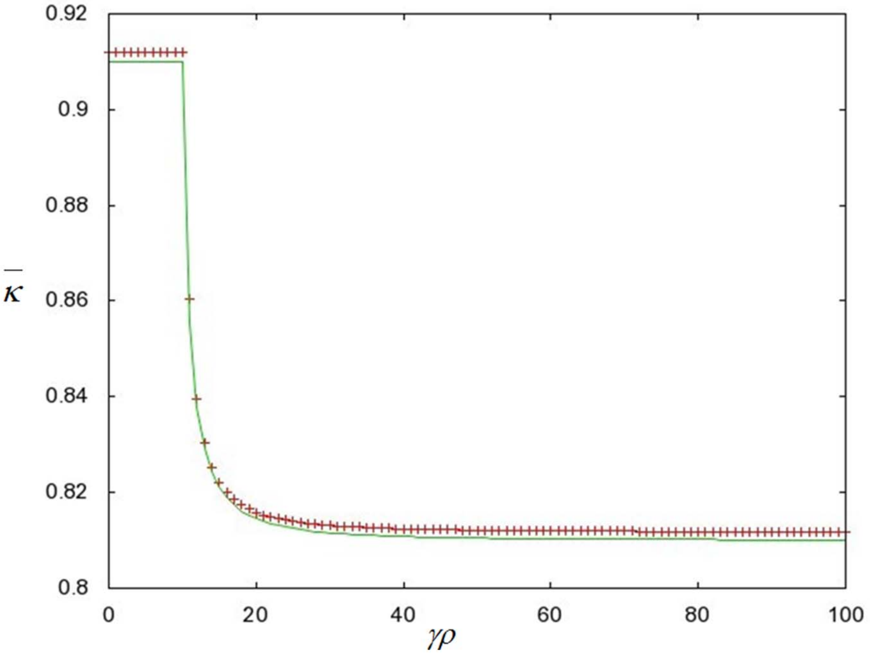

Figure 3. A plot of $\bar{\kappa}$ (the population mean fitness) versus $\gamma\rho$ (the second order reaction parameter of the conjugation rate). Parameter values are$\alpha_v = 0.8, \alpha_c = \alpha_r = 0.1, \kappa_D = 1.0, k_{+-} = 10.0, \mu = 0.1 (\alpha_c/\alpha_r/\alpha_v$ are the ratio of the genome which is dedicated to conjugation/ resistance/organism viability respectively, κ_D is the first-order death rate constant of the population that is caused by antibiotics, k_{+-} is the first order rate constant which describes the conjugators transition from the de-repressed to the repressed state and μ describes the probability of mutation for every replication of the entire genome). The solid line represents the analytical expression for $\bar{\kappa}$ obtained in the small-μ limit, while the pluses represent the values for $\bar{\kappa}$ obtained numerically. The numerical values for $\bar{\kappa}$ were obtained using fourth-order Runge-Kutta integration of the evolutionary dynamics equations. For $\gamma\rho \leq k_{+-}$, we used the initial condition $x_{-+} = 1.0, x^{+}_{++} = x^{-}_{++}, = x^{+}_{+-} = x^{-}_{+-} = x_{--} = 0.0$, while for $\gamma\rho > k_{+-}$ we used the initial condition $x^{+}_{++} = 1.0 - k_{+-}/(\gamma\rho), x^{-}_{++} = k_{+-}/(\gamma\rho), x^{+}_{+-} = x^{-}_{+-} = x_{-+} = x_{--} = 0.0$. We iterated for 100,000 time steps of size 0.001.
doi:10.1371/journal.pone.0096839.g003

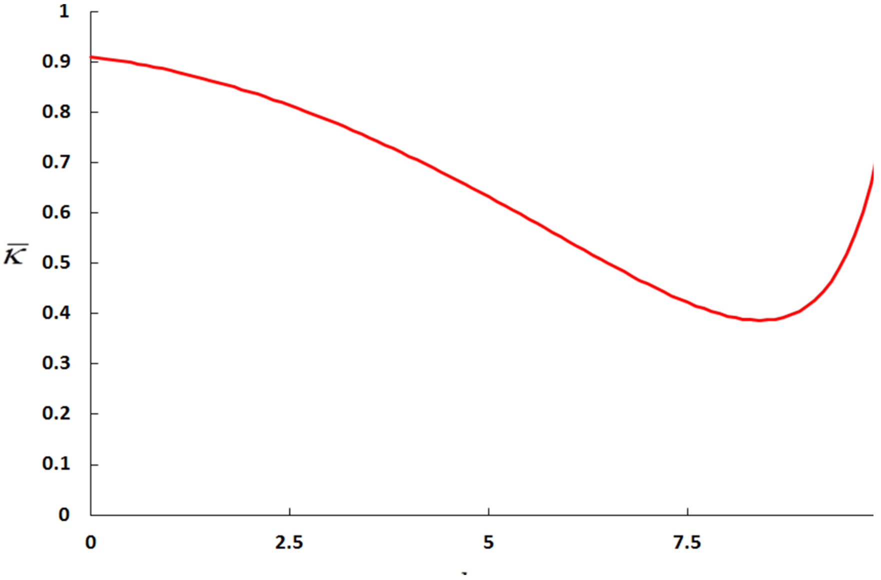

Figure 4. A graphical solution for the population mean fitness $\bar{\kappa}$ versus k_{+-} (the first order rate constant corresponding to the transition from the de-repressed to the repressed state of the conjugators). The values of $\bar{\kappa}$ were calculated from Eq. (12) with the parameter values: $\alpha_v = 0.8,\ \alpha_c = \alpha_r = 0.1,\ \kappa_D = 1.0,\ \gamma\rho = 10.0,\ \mu = 0.1.$
doi:10.1371/journal.pone.0096839.g004

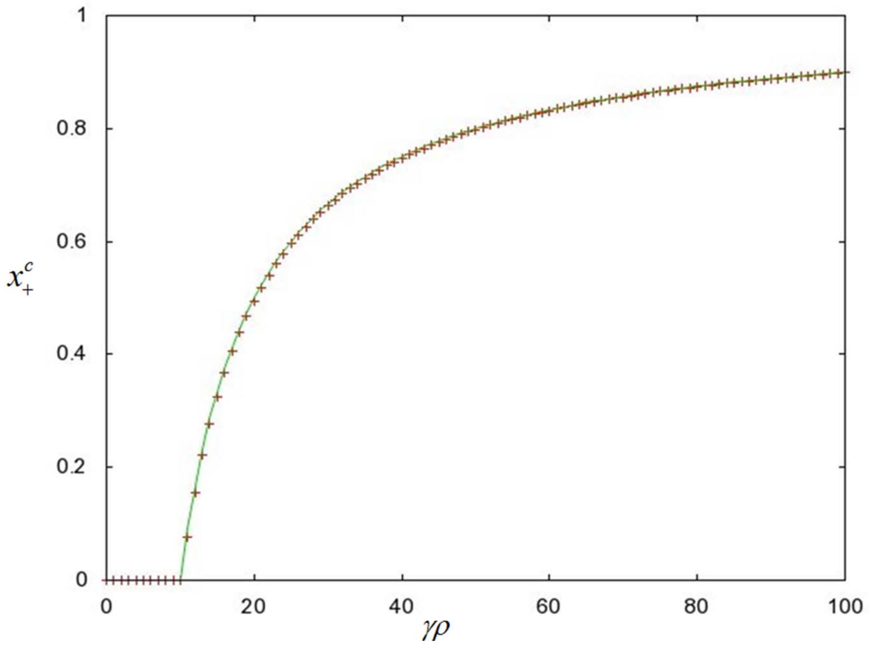

Figure 5. A plot of population fraction of conjugators $x_+^c = x_{++}^+ + x_{+-}^+$ versus the conjugation reaction parameter $\gamma\rho$. Parameter values are the same as those for figure 3, including the parameters for the Runge-Kutta integration. The solid line represents the zeroth-order analytical expression for x_+^c, while the pluses represent the values obtained numerically.
doi:10.1371/journal.pone.0096839.g005

found in sub-section F in the Materials and Methods and is illustrated by the graphical solution of $\bar{\kappa}$as a function of k_{+-} in Figure 4). For $k_{+-} < k_{+-,crit}$ the value of $\gamma\rho$ is big enough to lower the population mean fitness while for $k_{+-} > k_{+-,crit}$ the rate of transition from the de-repressed state to the repressed state is sufficiently large to decrease the difference in the mean fitness caused directly by conjugation. We should note that both the $k_{+-} \rightarrow \gamma\rho$ and $k_{+-} \rightarrow 0$ limits of $\bar{\kappa}$ give the same value of $\bar{\kappa} = 1 - \mu(\alpha_v + \alpha_r)$. Therefore, once the repression rate is greater than the value of $k_{+-,crit} = \gamma\rho + \kappa_D - \sqrt{\kappa_D(\gamma\rho + \kappa_D)}$, the decrease to the mean fitness caused by conjugation is mitigated to the point that it is effectively cancelled by repression.

Our study illustrates the critical role that repression is likely to have on regulating the process of conjugation and how it can

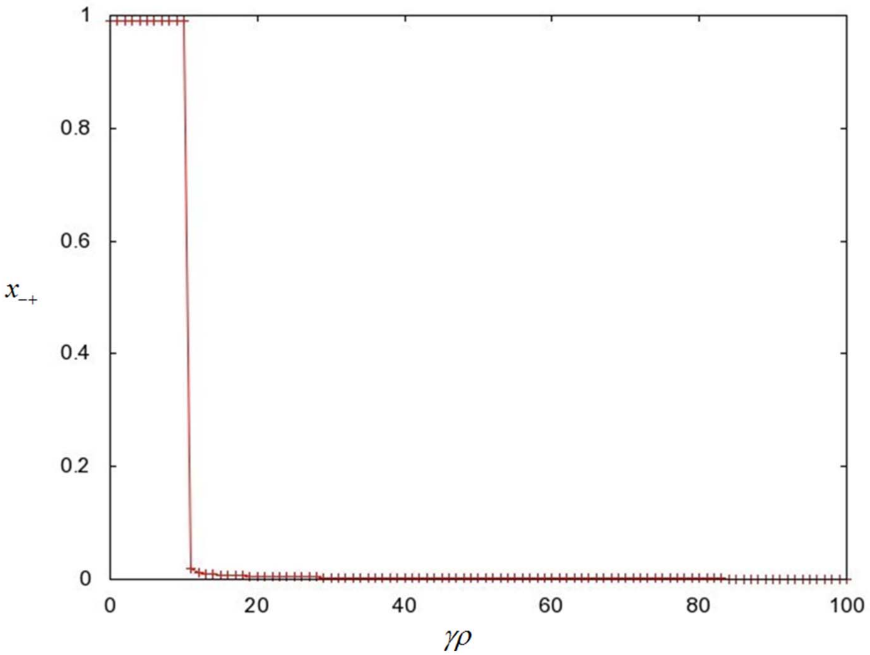

Figure 6. A plot of the population fraction of the non conjugating, antibiotic resistant bacteria sub-population x_{-+} versus the conjugation reaction parameter. The parameter values are the same as those for figure 3, including the parameters for the Runge-Kutta integration.
doi:10.1371/journal.pone.0096839.g006

negate the deleterious effect on the fitness of a population. Therefore, while conjugation is disadvantageous in a static environment, its negative effect on the fitness of a population is likely to become negligible if that population can sufficiently repress the plasmid and consequently, restore itself to its original fitness. This would suggest that the role of the bacteria in the actual host-parasite dynamics between the bacteria and the plasmid are much more complicated. While it is known that bacteria can obtain various advantageous traits from plasmids (including antibiotic resistance [3], degradation of unusual substances such as xylene, toluene, napthtalene and phenanthrene [45], resistance to heavy metals [46,47], and translation of Colicins [48]), they are also required to pay a fitness cost for hosting the plasmid in a static landscape. Previously, it was suggested that repression could reduce the energy costs for the host bacteria [49]. However, experimental results suggested that the amelioration of the fitness cost due to plasmid hosting was possible through evolution of a bacterial population [50]. Based on our results, we propose an alternative mechanism that allows bacteria to ameliorate this fitness cost by repressing their propensity towards conjugation and not only by vertical evolution. Interestingly, this strategy is likely to allow bacterial populations a significantly faster restoration pathway for their fitness loss (due to the maintenance costs of antibiotic drug resistance) then vertical gene transfer (i.e. evolution).

Our current model bears similarities to the Susceptible-Infected-Susceptible (SIS) [51–53] class of infectious disease models, where the "−" cells play the role of the Susceptibles (S) and the "+" cells play the role of the Infected (I). If the contact frequency between the S and the I population classes is sufficiently low, so that the I's revert back to the S state before having an opportunity to infect at least one additional S, then the fraction of I's in the population drops to a negligible fraction of the population. Above a critical contact frequency, however, the fraction of I's rises to a finite positive fraction of the population. Our model is in broad agreement with the behavior of actual conjugative plasmid systems, where it was observed that conjugative plasmids can spread in an infectious manner above a critical population density [38,54,55]. Typically this decrease in the population mean fitness is explained by the presence of Male Specific Phages (MSPs) [56], but in our model this factor is not accounted for, resulting in a decrease of the mean fitness that is a direct result of mutations that the plasmids go through when being replicated within the host bacteria.

We would like to also note recent studies that have suggested bacterial conjugation as a platform for the development of conjugation-based antibiotics [57]. In light of these studies, our results may have implications about the pharmaceutical limits that need to be considered for such antibiotic agents.

Finally, we believe that developing mathematical models that can correlate known data is important, as it indicates that the basic mathematical framework is reasonable, and may therefore serve as a foundation for building more sophisticated quantitative and predictive models. For future work, we will need to take into account more biological features of conjugation, such as regulation of plasmid copy number, MSPs and plasmid compatibility classes. Furthermore, we will need to move away from static landscapes and consider the role that conjugation has in allowing a population to adapt to new environments.

Materials and Methods

A. Proof That There Are No Non-resistant Organisms for $\mu=0,\kappa_D>0$

If we define x^r_+ to be the total fraction of resistant organisms, and x^r_- to be the total fraction of non-resistant organisms, then we have $x^r_+=x^+_{++}+x^-_{++}+x_{-+}$ and $x^r_-=x^+_{+-}+x^-_{+-}+x_{--}$. We then have that $\mu=0$,

$$\begin{aligned}
\frac{dx^+_{++}}{dt}&=[1-k_{+-}+\gamma\rho x^c_- -\overline{\kappa}(t)]x^+_{++}\\
\frac{dx^-_{++}}{dt}&=[1-\gamma\rho x^c_+ -\overline{\kappa}(t)]x^-_{++}+k_{+-}x^+_{++}\\
\frac{dx^+_{+-}}{dt}&=[1-\kappa_D-k_{+-}+\gamma\rho x^c_- -\overline{\kappa}(t)]x^+_{+-}\\
\frac{dx^-_{+-}}{dt}&=[1-\kappa_D-\gamma\rho x^c_+ -\overline{\kappa}(t)]x^-_{+-}+k_{+-}x^+_{+-}\\
\frac{dx_{-+}}{dt}&=[1-\gamma\rho x^c_+ -\overline{\kappa}(t)]x_{-+}\\
\frac{dx_{--}}{dt}&=[1-\kappa_D-\gamma\rho x^c_+ -\overline{\kappa}(t)]x_{--}
\end{aligned}\tag{8}$$

If we assume that the system converges to a stable steady-state, then we must have that $\overline{\kappa}(t)>1-k_{+-}+\gamma\rho x^c_-,1-\gamma\rho x^c_+$. If, for $\kappa_D>0$ there are non-resistant organisms in the population, then either x^+_{+-},x^-_{+-} or x_{--} are strictly positive. If $x^+_{+-}>0$, then we have $\overline{\kappa}=1-\kappa_D-k_{+-}+\gamma\rho x^c_-<1-k_{+-}+\gamma\rho x^c_-\Rightarrow\Leftarrow$. Therefore, $x^+_{+-}=0$, so either x^-_{+-} or $x_{--}$are >0, which gives $\overline{\kappa}=1-\kappa_D-\gamma\rho x^c_-<1-\gamma\rho x^c_-\Rightarrow\Leftarrow$.Thus, there are no non-resistant organisms in the population at steady-state for $\mu=0$.

B. Proof That $x^+_{0,++}=x^-_{0,++}=0,x_{0,-+}=1$ for $\gamma\rho<k_{+-}$

Let us suppose that $x^+_{++}>0$ for some$\gamma\rho<k_{+-},\mu$. Then the first line in Eq. (3) gives $\overline{\kappa}=2p_vp_cp_r-1-k_{+-}+\gamma\rho(2p_cp_r-1)x^c_-$. Now, assuming that our system converges to a stable steady-state, we also have from the the fifth line in Eq. (3) that$\overline{\kappa}\geq 2p_vp_r-1-\gamma\rho x^c_+$, and so $\gamma\rho-k_{+-}\geq\gamma\rho[x^c_+ +(2p_cp_r-1)x^c_-]-k_{+-}\geq 2p_vp_r(1-p_c)\geq 0\Rightarrow\Leftarrow$. Therefore, $x^+_{++}=0$ for $\gamma\rho<k_{+-},\mu$, which implies that $x^+_{0,++}=0$.

Suppose then that, $x^-_{++}>0$for $\gamma\rho<k_{+-},\mu>0$. Then from Eq. (3) we have$\overline{\kappa}=2p_vp_cp_r-1-\gamma\rho x^c_+<2p_vp_r-1-\gamma\rho x^c_+\Rightarrow\Leftarrow$, hence $x^-_{++}=0$ for $\gamma\rho<k_{+-},\mu>0$.

Taking the limit as $\mu\to 0$, we obtain $x^-_{0,++}=0$for $\gamma\rho<k_{+-}$. Now, when $\mu=0$, we have $x^+_{0,++}+x^-_{0,++}+x_{0,-+}=1$, and so for we obtain $x_{0,-+}=1$.

C. Proof That $x^+_{0,++}=1-k_{+-}/(\gamma\rho),x^-_{0,++}=k_{+-}/(\gamma\rho)$, $x_{0,-+}=0$ for $\gamma\rho>k_{+-}$

Given some $\gamma\rho>k_{+-}$, suppose that $x^+_{0,++}=0$. Then since that are no non-resistant organisms for$\mu=0$, we have that $x^c_{0,+}=x^c_{0,++}=0$ and $x^c_{0,-}=1$. This implies that$\overline{\kappa}\geq 1-k_{+-}+\gamma\rho>1\Rightarrow\Leftarrow$, and so we must have that $x^+_{0,++}>0$. This gives, $1=\overline{\kappa}=1-k_{+-}+\gamma\rho(x^-_{0,++}+x_{0,-+})\Rightarrow x^-_{0,++}+x_{0,-+}=k_{+-}/(\gamma\rho)$, and so $x^-_{0,++}=1-k_{+-}/(\gamma\rho)$.

Now, the fifth line of Eq. (3) gives,

$$0=-\gamma\rho x^{+}_{0,++}x_{0,-+} \tag{9}$$

from which it follows that $x_{0,-+}=0$, and so $x^{-}_{0,++}=k_{+-}/(\gamma\rho)$.

D. Derivation of the Small-μ Expressions for $x^{\pm}_{+-}, x_{--}$

For small μ, we may write the first-order expressions for $dx^{\pm}_{+-}/dt$ and dx_{--}/dt. We obtain,

$$\begin{aligned}0&=[-\kappa_D-k_{+-}+\gamma\rho(x^{-}_{0,++}+x_{0,-+})]x^{+}_{+-}\\&+2(1-p_r)x^{+}_{0,++}(1+\gamma\rho(x^{-}_{0,++}+x_{0,-+}))\\0&=-[\kappa_D+\gamma\rho x^{+}_{0,++}]x^{-}_{+-}+k_{+-}x^{+}_{+-}+2(1-p_r)x^{-}_{0,++}\\0&=-[\kappa_D+\gamma\rho x^{+}_{0,++}]x_{--}+2(1-p_r)x_{0,-+}\end{aligned} \tag{10}$$

For $\gamma\rho<k_{+-}$, we have $x^{+}_{0,++}=x^{-}_{0,++}=0$, and $x_{0,-+}=1$, which implies $x^{+}_{+-}=x^{-}_{+-}=0$, and.

$x_{--}=x^{r}_{-}=2(1-p_r)/\kappa_D$, thus $\bar{\kappa}=2p_v-1-\kappa_D x^{r}_{-}=2p_v-1-2(1-p_r)=1-(\alpha_v+\alpha_r)\mu$.

For $\gamma\rho>k_{+-}$ we have $x^{+}_{0,++}=1-k_{+-}/\gamma\rho, x^{-}_{0,++}=k_{+-}/\gamma\rho$, and $x_{0,-+}=0$. This gives,

$$\begin{aligned}x^{+}_{+-}&=\frac{2(1-p_r)}{\kappa_D}(1-\frac{k_{+-}}{\gamma\rho})(1+k_{+-})\\x^{-}_{+-}&=\frac{2(1-p_r)}{\kappa_D}\frac{\frac{k_{+-}}{\kappa_D}(1-\frac{k_{+-}}{\gamma\rho})(1+k_{+-})+\frac{k_{+-}}{\gamma\rho}}{1-\frac{k_{+-}}{\kappa_D}+\frac{\gamma\rho}{\kappa_D}}\\x_{--}&=0,\end{aligned} \tag{11}$$

and so the mean fitness at steady-state is given by,

$$\bar{\kappa}=1-\mu\left(\alpha_v+\alpha_r[(1-\frac{k_{+-}}{\gamma\rho})(1+k_{+-})+\frac{\frac{k_{+-}}{\kappa_D}(1-\frac{k_{+-}}{\gamma\rho})(1+k_{+-})+\frac{k_{+-}}{\gamma\rho}}{1-\frac{k_{+-}}{\kappa_D}+\frac{\gamma\rho}{\kappa_D}}]\right) \tag{12}$$

E. Derivation of $(\gamma\rho)_{crit}$ for $\gamma\rho>k_{+-}$

In order to find the critical value of $\gamma\rho$, we compute the derivative of Eq. (12) with respect to$\gamma\rho$and derive the following expression:

$$\frac{d\bar{\kappa}}{d(\gamma\rho)}=\frac{d}{d(\gamma\rho)}\left\{1-\mu(\alpha_v+\alpha_r\left[\frac{\frac{k_{+-}}{\kappa_D}(1-\frac{k_{+-}}{\gamma\rho})(1+k_{+-})+\frac{k_{+-}}{\gamma\rho}}{1-\frac{k_{+-}}{\kappa_D}+\frac{\gamma\rho}{\kappa_D}}\right])\right\}=-\mu\alpha_r\frac{\kappa_D k_{+-}{}^2(2\gamma\rho+\kappa_D-k_{+-})}{(\gamma\rho)^2(\gamma\rho+\kappa_D-k_{+-})^2} \tag{13}$$

Equating this term to zero allows us to find $(\gamma\rho)_{crit}$:

$$\begin{aligned}&\frac{d\bar{\kappa}}{d(\gamma\rho)}=-\mu\alpha_r\frac{\kappa_D k_{+-}{}^2(2\gamma\rho+\kappa_D-k_{+-})}{(\gamma\rho)^2(\gamma\rho+\kappa_D-k_{+-})^2}=0\\&\rightarrow 2\gamma\rho+\kappa_D-k_{+-}=0\\&\rightarrow(\gamma\rho)_{crit}=\frac{1}{2}(k_{+-}-\kappa_D)\end{aligned} \tag{14}$$

F. Derivation of $k_{+-,crit}$

In order to find the critical value of k_{+-}we take the derivative of Eq. (12) with respect to $k_{+-}o$and get the following expression:

$$\begin{aligned}\frac{d\bar{\kappa}}{dk_{+-}}&=\frac{d}{dk_{+-}}\left\{1-\mu(\alpha_v+\alpha_r\left[(1-\frac{k_{+-}}{\gamma\rho})(1+k_{+-})+\frac{\frac{k_{+-}}{\kappa_D}(1-\frac{k_{+-}}{\gamma\rho})(1+k_{+-})+\frac{k_{+-}}{\gamma\rho}}{1-\frac{k_{+-}}{\kappa_D}+\frac{\gamma\rho}{\kappa_D}}\right])\right\}\\&=-\frac{\alpha_r\mu(\gamma\rho+\kappa_D)\left[(\gamma\rho)^2+\gamma\rho(\kappa_D-2k_{+-})-k_{+-}(2\kappa_D-k_{+-})\right]}{\gamma\rho(\gamma\rho+\kappa_D-k_{+-})^2}\end{aligned} \tag{15}$$

By equating this expression to zero we get the following quadratic equation for k_{+-}:

$$\begin{aligned}&\alpha_r\mu(\gamma\rho+\kappa_D)\left[(\gamma\rho)^2+\gamma\rho(\kappa_D-2k_{+-})-k_{+-}(2\kappa_D-k_{+-})\right]=0\\&(\gamma\rho)^2+\gamma\rho(\kappa_D-2k_{+-})-k_{+-}(2\kappa_D-k_{+-})=0\\&\gamma\rho\kappa_D-2\gamma\rho k_{+-}-2\kappa_D k_{+-}+k_{+-}{}^2+(\gamma\rho)^2=0\\&k_{+-}{}^2-k_{+-}(2\gamma\rho+2\kappa_D)+\gamma\rho(\gamma\rho+\kappa_D)=0,\end{aligned} \tag{16}$$

which allows us to find the values for k_{+-} that solve the aforementioned equation:

$$\begin{aligned}k_{+-}&=\frac{2\gamma\rho+2\kappa_D\pm\sqrt{(-2\gamma\rho-2\kappa_D)^2-4\cdot1\cdot\gamma\rho(\gamma\rho+\kappa_D)}}{2\cdot1}=\\&\frac{2\gamma\rho+2\kappa_D\pm\sqrt{4\gamma\rho^2+8\gamma\rho\cdot\kappa_D+4\kappa_D{}^2-4\gamma\rho^2-4\gamma\rho\cdot\kappa_D}}{2}=\\&\gamma\rho+\kappa_D\pm\sqrt{\kappa_D(\gamma\rho+\kappa_D)}\end{aligned} \tag{17}$$

Once we take into account the singularity of $\bar{\kappa}$ at $1-\frac{k_{+-}}{\kappa_D}+\frac{\gamma\rho}{\kappa_D}$ we are left only with the following:

$$k_{+-,crit}=\gamma\rho+\kappa_D-\sqrt{\kappa_D(\gamma\rho+\kappa_D)} \tag{18}$$

Note that one can compute that.

$$\frac{d^2\bar{\kappa}}{dk_{+-}^2}=\frac{2\alpha_r\cdot\kappa_D\cdot\mu(\gamma\rho+\kappa_D)^2}{\gamma\rho(\gamma\rho+\kappa_D-k_{+-})^3}$$
$$\frac{d^2\bar{\kappa}\left[\gamma\rho+\kappa_D-\sqrt{\kappa_D(\gamma\rho+\kappa_D)}\right]}{dk_{+-}{}^2}=\frac{2\alpha_r\cdot\kappa_D\cdot\mu(\gamma\rho+\kappa_D)^2}{\gamma\rho(\gamma\rho+\kappa_D-k_{+-})^3}$$
$$=\frac{2\alpha_r\cdot\kappa_D\cdot\mu(\gamma\rho+\kappa_D)^2}{\gamma\rho(\gamma\rho+\kappa_D-\left[\gamma\rho+\kappa_D-\sqrt{\kappa_D(\gamma\rho+\kappa_D)}\right])^3}= \quad (19)$$
$$\frac{2\alpha_r\cdot\kappa_D\cdot\mu(\gamma\rho+\kappa_D)^2}{\gamma\rho(\gamma\rho+\kappa_D-\gamma\rho-\kappa_D+\sqrt{\kappa_D(\gamma\rho+\kappa_D)})^3}=\frac{2\alpha_r\cdot\kappa_D\cdot\mu(\gamma\rho+\kappa_D)^2}{\gamma\rho(\sqrt{\kappa_D(\gamma\rho+\kappa_D)})^3}$$

If we take into account the positivity of $\gamma\rho,\kappa_D,\alpha_r,\mu$, we see that $\frac{2\alpha_r\cdot\kappa_D\cdot\mu(\gamma\rho+\kappa_D)^2}{\gamma\rho(\sqrt{\kappa_D(\gamma\rho+\kappa_D)})^3}>0$, and so the critical value $\gamma\rho+\kappa_D-\sqrt{\kappa_D(\gamma\rho+\kappa_D)}$ is indeed a (local) minimum value of $\bar{\kappa}$.

Acknowledgments

This paper was completed after the untimely death of Professor Emmanuel David Tannenbaum (of blessed memory) in May 2012, the author accepts all responsibility for the integrity of the data, analysis and results as published. Emmanuel was an inspiring adviser, a great friend and an exceptional person in all aspects of his life. His kindness, brilliance, and humanity will always be remembered and treasured by those of us who had the honor of knowing and working with him. The author would also like to thank Dr. Nathaniel Wagner from Ben-Gurion University for the productive discussions, criticism and advice.

Author Contributions

Conceived and designed the experiments: EDT YR. Performed the experiments: EDT YR. Analyzed the data: EDT YR. Contributed reagents/materials/analysis tools: EDT YR. Wrote the paper: EDT YR.

References

1. Gogarten JP, Townsend JP (2005) Horizontal gene transfer, genome innovation and evolution. Nat Rev Microbiol 3: 679–687.
2. Ochman H, Lawrence JG, Groisman EA (2000) Lateral gene transfer and the nature of bacterial innovation. Nature 405: 299–304.
3. Tatum EL, Lederberg J (1947) Gene Recombination in the Bacterium Escherichia coli. J Bacteriol 53: 673–684.
4. de la Cruz F, Davies J (2000) Horizontal gene transfer and the origin of species: lessons from bacteria. Trends Microbiol 8: 128–133.
5. Roberts MC (2008) Update on macrolide-lincosamide-streptogramin, ketolide, and oxazolidinone resistance genes. FEMS Microbiol Lett 282: 147–159.
6. Hanssen AM, Ericson Sollid JU (2006) SCCmec in staphylococci: genes on the move. FEMS Immunol Med Microbiol 46: 8–20.
7. Robicsek A, Jacoby GA, Hooper DC (2006) The worldwide emergence of plasmid-mediated quinolone resistance. Lancet Infect Dis 6: 629–640.
8. Weldhagen GF (2004) Integrons and β - lactamases–a novel perspective on resistance. International Journal of Antimicrobial Agents 23: 556.
9. Park JM, Deem MW (2007) Phase diagrams of quasispecies theory with recombination and horizontal gene transfer. Phys Rev Lett 98: 058101.
10. Sun J, Deem MW (2007) Spontaneous emergence of modularity in a model of evolving individuals. Phys Rev Lett 99: 228107.
11. Munoz E, Park JM, Deem MW (2008) Quasispecies theory for horizontal gene transfer and recombination. Phys Rev E Stat Nonlin Soft Matter Phys 78: 061921.
12. Cohen E, Kessler DA, Levine H (2005) Recombination dramatically speeds up evolution of finite populations. Phys Rev Lett 94: 098102.
13. Tannenbaum ED, Shakhnovich EI (2005) Semiconservative replication, genetic repair, and many - gened genomes: Extending the quasispecies paradigm to living systems. Physics of Life Reviews 2: 290.
14. Dixit NM, Srivastava P, Vishnoi NK (2012) A finite population model of molecular evolution: theory and computation. J Comput Biol 19: 1176–1202.
15. Brumer Y, Michor F, Shakhnovich EI (2006) Genetic instability and the quasispecies model. J Theor Biol 241: 216–222.
16. Saakian DB, Hu CK (2006) Exact solution of the Eigen model with general fitness functions and degradation rates. Proc Natl Acad Sci U S A 103: 4935–4939.
17. Richter H (2014) Frontiers of Fitness Landscapes: A Summary of Open Questions. In: Richter H, Engelbrecht A, editors. Recent Advances in the Theory and Application of Fitness Landscapes: Springer Berlin Heidelberg. 529–544.
18. Gorodetsky P, Tannenbaum E (2008) Effect of mutators on adaptability in time-varying fitness landscapes. Phys Rev E Stat Nonlin Soft Matter Phys 77: 042901.
19. Tannenbaum ED, Shakhnovich EI (2004) Solution of the quasispecies model for an arbitrary gene network. Phys Rev E Stat Nonlin Soft Matter Phys 70: 021903.
20. Itan E, Tannenbaum E (2012) Effect of chromosomal instability on the mutation-selection balance in unicellular populations. PLoS One 7: e26513.
21. Campos PRA, Fontanari JF (1998) Finite-size scaling of the quasispecies model. Physical Review E 58: 2664–2667.
22. Gogarten JP, Doolittle WF, Lawrence JG (2002) Prokaryotic evolution in light of gene transfer. Mol Biol Evol 19: 2226–2238.
23. Brown JR (2003) Ancient horizontal gene transfer. Nat Rev Genet 4: 121–132.
24. Doolittle WF (1999) Phylogenetic classification and the universal tree. Science 284: 2124–2129.
25. Rivera MC, Lake JA (2004) The ring of life provides evidence for a genome fusion origin of eukaryotes. Nature 431: 152–155.
26. Cruz F, Lanka E (1998) Function of the Ti-Plasmid Vir Proteins: T-Complex Formation and Transfer to the Plant Cell. In: Spaink H, Kondorosi A, Hooykaas PJ, editors. The Rhizobiaceae: Springer Netherlands. 281–301.
27. Grillot-Courvalin C, Goussard S, Huetz F, Ojcius DM, Courvalin P (1998) Functional gene transfer from intracellular bacteria to mammalian cells. Nat Biotechnol 16: 862–866.
28. Russi S, Boer R, Coll M (2008) Molecular machinery for DNA translocation in bacterial conjugation. In: Lipps G, editor. Plasmids: Current Research and Future Trends: Caister Academic Press,Cambridge, UK. 183–214.
29. Carattoli A (2013) Plasmids and the spread of resistance. Int J Med Microbiol 303: 298–304.
30. Andam CP, Fournier GP, Gogarten JP (2011) Multilevel populations and the evolution of antibiotic resistance through horizontal gene transfer. FEMS Microbiol Rev 35: 756–767.
31. Davies J (1994) Inactivation of antibiotics and the dissemination of resistance genes. Science 264: 375–382.
32. Salyers AA, Shoemaker NB, Stevens AM, Li LY (1995) Conjugative transposons: an unusual and diverse set of integrated gene transfer elements. Microbiol Rev 59: 579–590.
33. Mazel D, Davies J (1999) Antibiotic resistance in microbes. Cell Mol Life Sci 56: 742–754.
34. Ghigo JM (2001) Natural conjugative plasmids induce bacterial biofilm development. Nature 412: 442–445.
35. Frost LS, Koraimann G (2010) Regulation of bacterial conjugation: balancing opportunity with adversity. Future Microbiol 5: 1057–1071.
36. Raz Y, Tannenbaum E (2010) The influence of horizontal gene transfer on the mean fitness of unicellular populations in static environments. Genetics 185: 327–337.
37. Kamp C, Wilke CO, Adami C, Bornholdt S (2002) Viral evolution under the pressure of an adaptive immune system: Optimal mutation rates for viral escape. Complexity 8: 28–33.
38. Lundquist PD, Levin BR (1986) Transitory derepression and the maintenance of conjugative plasmids. Genetics 113: 483–497.
39. Norman A, Hansen LH, Sorensen SJ (2009) Conjugative plasmids: vessels of the communal gene pool. Philos Trans R Soc Lond B Biol Sci 364: 2275–2289.
40. Novick RP (1987) Plasmid incompatibility. Microbiol Rev 51: 381–395.
41. Park K, Han E, Paulsson J, Chattoraj DK (2001) Origin pairing ('handcuffing') as a mode of negative control of P1 plasmid copy number. EMBO J 20: 7323–7332.
42. Uhlin BE, Nordstrom K (1975) Plasmid incompatibility and control of replication: copy mutants of the R-factor R1 in Escherichia coli K-12. J Bacteriol 124: 641–649.
43. Turner PE, Cooper VS, Lenski RE (1998) Tradeoff between horizontal and vertical modes of transmission in bacterial plasmids. Evolution: 315–329.
44. Crotty S, Cameron CE, Andino R (2001) RNA virus error catastrophe: Direct molecular test by using ribavirin. Proceedings of the National Academy of Sciences 98: 6895–6900.
45. Sanseverino J, Applegate BM, King JM, Sayler GS (1993) Plasmid-mediated mineralization of naphthalene, phenanthrene, and anthracene. Appl Environ Microbiol 59: 1931–1937.

46. Mergeay M, Nies D, Schlegel HG, Gerits J, Charles P, et al. (1985) Alcaligenes-Eutrophus Ch34 Is a Facultative Chemolithotroph with Plasmid-Bound Resistance to Heavy-Metals. Journal of Bacteriology 162: 328–334.
47. Silver S, Misra TK (1988) Plasmid-mediated heavy metal resistances. Annu Rev Microbiol 42: 717–743.
48. Cascales E, Buchanan SK, Duche D, Kleanthous C, Lloubes R, et al. (2007) Colicin biology. Microbiol Mol Biol Rev 71: 158–229.
49. Anderson ES (1968) The ecology of transferable drug resistance in the enterobacteria. Annu Rev Microbiol 22: 131–180.
50. Dahlberg C, Chao L (2003) Amelioration of the cost of conjugative plasmid carriage in Eschericha coli K12. Genetics 165: 1641–1649.
51. Weiss GH, Dishon M (1971) On the asymptotic behavior of the stochastic and deterministic models of an epidemic. Mathematical Biosciences 11: 261.
52. Nåsell I (1996) The Quasi-Stationary Distribution of the Closed Endemic SIS Model. Advances in Applied Probability 28: 895.
53. Kryscio RJ, Lefevre C (1989) On the Extinction of the S-I-S Stochastic Logistic Epidemic. Journal of Applied Probability 26: 685–694.
54. Willetts NS (1974) The kinetics of inhibition of Flac transfer by R100 in E. coli. Mol Gen Genet 129: 123–130.
55. Broda P (1975) Transience of the donor state in an Escherichia coli K12 strain carrying a repressed R factor. Mol Gen Genet 138: 65–69.
56. Caro LG, Schnos M (1966) The attachment of the male-specific bacteriophage F1 to sensitive strains of Escherichia coli. Proc Natl Acad Sci U S A 56: 126–132.
57. Filutowicz M, Burgess R, Gamelli RL, Heinemann JA, Kurenbach B, et al. (2008) Bacterial conjugation-based antimicrobial agents. Plasmid 60: 38–44.
